# Design, synthesis, and antiproliferative screening of new quinoline derivatives bearing a *cis*-vinyl triamide motif as apoptosis activators and EGFR-TK inhibitors†

**DOI:** 10.1039/d4ra04915b

**Published:** 2024-08-07

**Authors:** Hany M. Abd El-Lateef, Ahmed Gaafar Ahmed Gaafar, Arwa Sultan Alqahtani, Aamal A. Al-Mutairi, Dalal Sulaiman Alshaya, Fahmy Gad Elsaid, Eman Fayad, N. A. Farouk

**Affiliations:** a Department of Chemistry, College of Science, King Faisal University Al-Ahsa 31982 Saudi Arabia hmahmed@kfu.edu.sa; b Department of Chemistry, Faculty of Science, Sohag University Sohag 82524 Egypt; c Department of Pharmacology and Toxicology, Faculty of Pharmacy, Port Said University Port Said Egypt; d Department of Chemistry, College of Science, Imam Mohammad Ibn Saud Islamic University(IMSIU) P.O. Box 90950 Riyadh 11623 Saudi Arabia; e Department of Biology, College of Science, Princess Nourah bint Abdulrahman University P.O. Box 84428 Riyadh 11671 Saudi Arabia; f Department of Biology, College of Science, King Khalid University PO Box 960 Abha 61421 Asir Saudi Arabia; g Department of Biotechnology, College of Sciences, Taif University P.O. Box 11099 Taif 21944 Saudi Arabia; h Department of Chemistry, Faculty of Science, Port Said University Port Said Egypt nesmafarouk566@gmail.com

## Abstract

In this work, a congeneric set of quinoline-tethered *cis*-vinyl triamide hybrids was prepared and evaluated as EGFR tyrosine kinase inhibitors for the management of breast cancer. All of the prepared hybrids were evaluated for their antiproliferative effect against the breast MCF-7 cell line. Among the tested hybrids, compound 6f displayed the most potent antiproliferative activity with an IC_50_ value of 1.87 μM compared to STU (IC_50_ = 13.71 μM) as the standard reference. The most promising hybrid, 6f, was found to induce cellular cycle arrest at the G1 phase. Furthermore, the molecular mechanism of this hybrid revealed its ability to induce cellular apoptosis *via* the mitochondrial-dependent apoptotic pathway. Compound 6f decreased MCF-7 cells' MMP compared to the controls (percentage change value of 57.93%). Further investigation of the selective compound 6f showed that it can inhibit EGFR tyrosine kinase.

## Abstract 2

A set of novel quinoline tethered *cis*-vinyl triamides derivatives has been designed, synthesized and screened for *in vitro* cytotoxicity against the MCF-7 breast adenocarcinoma cell line.

## Graphical abstract

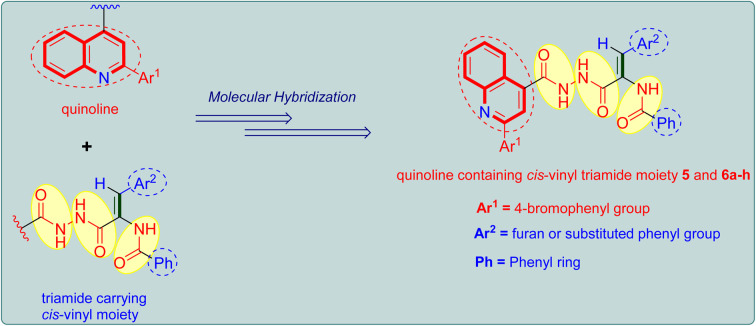

## 1. Introduction

Cancer remains the leading cause of death in the world, second only to heart disease.^1,2^ Cancer itself is a dynamic, complex cellular network of uncontrolled growth.^3^ Of all cancer subtypes, breast cancer is one of the most deadly, and also consistently beckons the largest number of new diagnoses among women.^4,5^ Clearly, there is a serious unmet demand for therapies targeting this cancer subtype.^6,7^ Effective chemotherapeutic treatments with minimal side effects are urgently needed, particularly in the face of the increasing prevalence of drug-resistant tumors.^8^ Therapies based on targeted biological target are intended to be less toxic than conventional chemotherapy.^9,10^

The epidermal growth factor receptor (EGFR), being one of the most prominent protein kinases, plays a vital role in a series of cellular processes during the life cycle of the cell, such as the regulation of cell migration and cell division.^11,12^ Multiple prospective clinical trials were initiated to test the efficacy of EGFR-targeted therapy *versus* standard chemotherapy.^13^ Clinical trials revealed definite evidence of the superiority of EGFR tyrosine kinase inhibitors over standard chemotherapeutic regimens.^14^ Targeted inhibitors of EGFR signalling inhibited ligand-induced EGFR auto-phosphorylation and attenuated downstream signalling responsible for proliferation and survival of the cells.^15,16^ The inhibition of EGFR signalling leads, in most cases, to cell cycle arrest and/or drug-induced apoptosis.^17,18^ Therefore, EGFR has been regarded as an attractive target for the establishment of chemotherapeutic drugs for many cancers.^19^ Accordingly, the discovery of new EGFR has attracted a great deal of attention in recent years.^20^

**Fig. 1:**
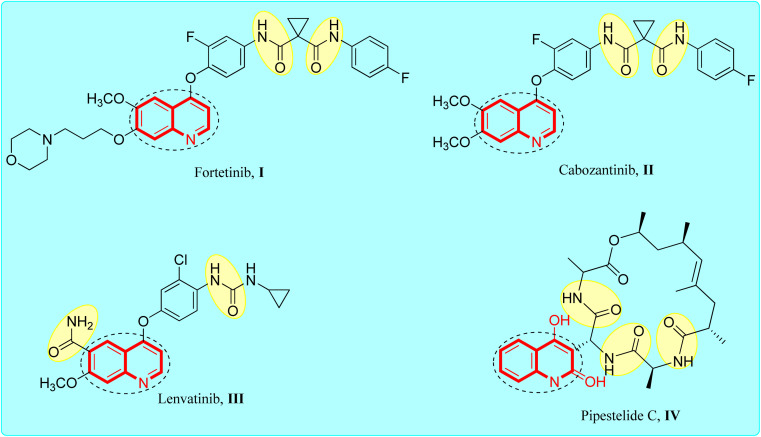
Reference quinoline-amide tethered anticancer agents.

Quinolines are a distinct class of fused bicyclic hetero-aromatic core compounds that have developed into a very popular research topic.^21–23^ Several quinoline-based compounds are known to have chemical, biological and therapeutic applications.^24,25^ They were found to exhibit anticancer, antiviral and antimicrobial effects.^26–28^ The quinoline nucleus is also an integral component of several anticancer drugs, which have revolutionized the therapy of cancer disease.^29–31^ The anti-cancer activity seems to be due to a variety of mechanisms include inhibition of cellular promoting factors such as tubulin polymerization and topoisomerase.^32,33^ Recently, several research studies have described quinoline derivatives with anticancer efficacy *via* inhibition of kinases and inhibition of the anti-death Bcl-2 family of proteins^34,35^ (Fig. 1).

The amide pharmacophore is an important framework for developing drugs and discovery.^36^ According to medicinal chemistry sources, one or more amide bonds serve as the basic component in more than 25% of natural and synthetic drugs available in the market.^37^ The amide functional group enhances the anticancer activity by making molecules more polar as well as forming a hydrogen bond acceptor–donor domain with the target receptor.^38^ Therefore, the introduction of an amide motif bearing a *cis*-vinyl group into the quinoline core is likely to significantly influence biological activity.^39^

**Fig. 2:**
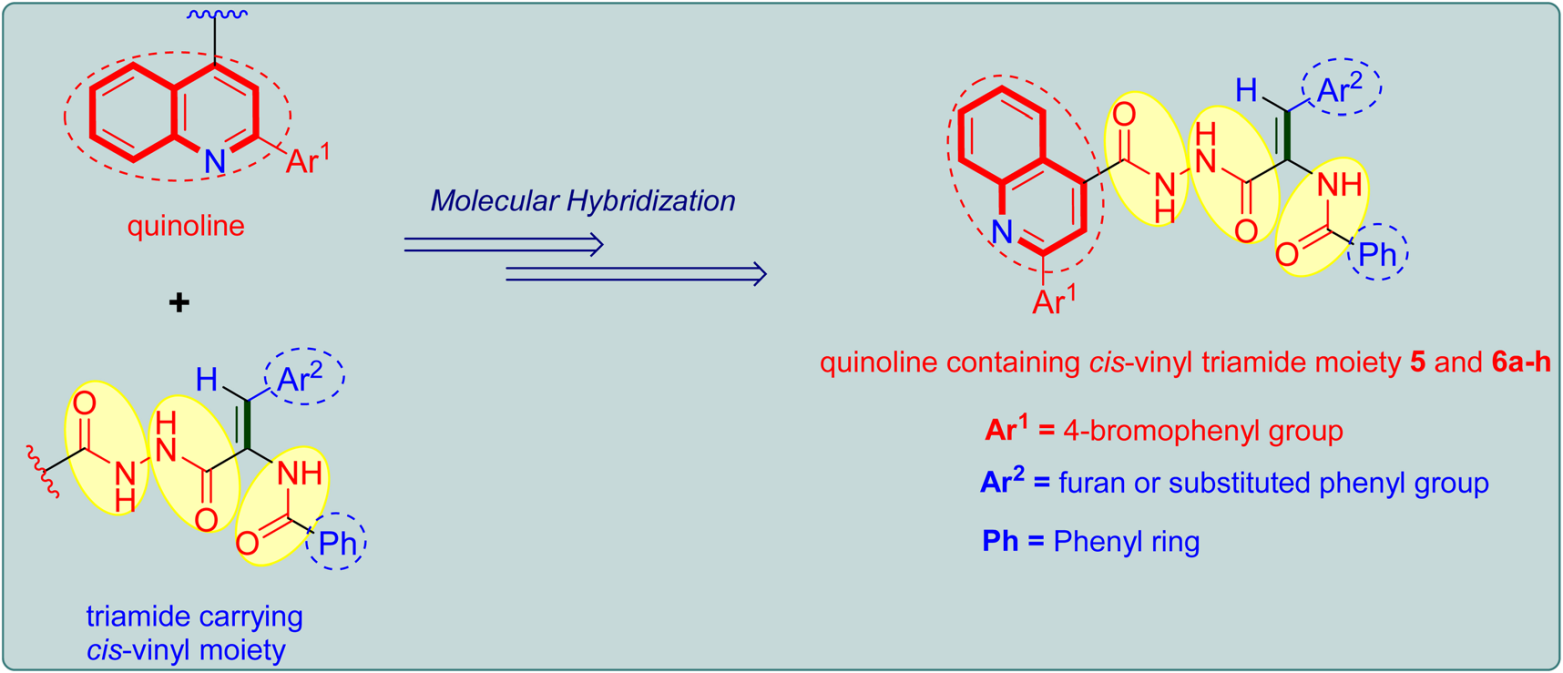
Design strategy adopted for the design of target quinoline tethered *cis*-vinyl triamides 5 and 6a–h.

The aforementioned intriguing findings, combined with our ongoing quest for more potent anticancer agents, led to the molecular hybridization of the quinoline core and the bioactive triamide motif bearing a *cis*-vinyl group in order to integrate them into a single molecular framework and achieve a new hybrid that could have potential antiproliferative activity (Fig. 2). The study's goal is to look into the benefits of such hybridization in terms of predicted biological activity and to see whether this resulted in better biological activity for the produced hybrids. Lastly, we used *in vitro* tests to assess the antiproliferative action of the prepared quinoline-*cis*-vinyl triamide hybrids, as well as FACS and ELISA methods to determine the mechanism of cellular action.

## 2. Results and discussion

### 2.1. Chemistry

**Scheme 1:**
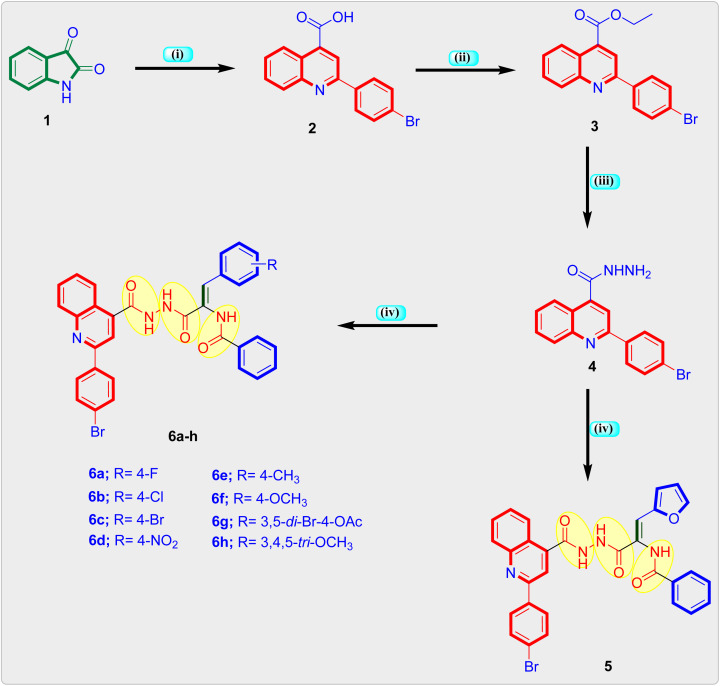
Global synthesis of quinoline-*cis*-vinyl triamide hybrids 5 and 6a–h. Reagents: (i) 4-Br–C_6_H_4_COCH_3_, 33% KOH, EtOH, reflux 24 h, yield = 89%; (ii) H_2_SO_4_, EtOH, reflux 12 h, yield = 80%; (iii) NH_2_NH_2_·H_2_O, EtOH, reflux 4 h, yield = 77%; (iv) respective methyl 3-aryl-2-(benzamido)-2-propenoate, EtOH, reflux 20–22 h, yield: 64–78%.

The synthetic route used in the preparation of the intermediate and final quinoline based triamide compounds is depicted in Scheme 1. In the initial step appropriate 2-(4-bromoquinoline)-4-carboxylic acid 2 was obtained from reaction of isatin 1 and 4-bromoacetophenone in the presence of 33% KOH in pure ethanol by refluxing which was then heated to reflux in pure ethanol in the presence of concentrated sulphuric acid to obtain ethyl quinoline-4-carboxylate 3.^26^ This is followed by hydrazinolysis with pure hydrazine hydrate in boiling ethanol to furnish the key intermediate quinoline-4-carbohydrazide compound 4.^40,41^ Lastly, final quinolone-linked triamide derivatives 5 and 6a–h were obtained by the reaction of quinoline-4-carbohydrazide 4 and respective methyl 3-aryl-2-(benzamido)-2-propenoate derivative in freshly molten sodium acetate and glacial acetic acid by refluxing and yielding 64–78%. The formation of the quinoline linked triamide compounds 5 and 6a–h in general were authenticated by ^1^H-NMR, ^13^C-NMR and elemental analysis. ^1^H-NMR spectra of the final quinoline-like triamide molecules displayed three broad singlets at range *δ* 10.89–9.99 ppm ascribed to the three amide protons and singlet peak at *δ* 7.48–7.37 ppm range assigned to olefinic (=CH) proton. The characteristic doublet signal at about *δ* 8.46–8.42 ppm is attributed to the C8–H of the quinoline motif. In addition, the protons of C7–H and C6–H of quinoline motif appeared as two triplet signals with one proton each in the range at *δ* 7.88–7.72 and 7.72–7.63 ppm, respectively.

The ^13^C-NMR spectra of the final compounds displayed signals for aromatic and olefinic carbon atoms were observed at around *δ* 153.09–107.71 ppm, whereas signals for aliphatic carbon atoms in compounds 6f, 6e, 6g and 6h were observed at around *δ* 60.54–20.65. The characteristic C2 carbon of quinoline moiety was identified in the range *δ* 155.03–155.00 ppm. Furthermore, the carbonyl carbons of the three amide functions were seen at about *δ* 168.85–164.45 ppm providing a substantial argument in support of the compounds' ascribed structures. Each of the target compounds was characterized using the melting point technique, and their purity was verified using the TLC method. The newly prepared quinoline-triamide molecules' analytical and spectral data (^1^H-NMR and ^13^C-NMR spectra) agreed fully with the suggested structures.

### 2.2. Biology

#### 2.2.1. Antiproliferative activity

The antiproliferative potential of the newly prepared quinoline tethered *cis*-vinyl triamide derivatives 5 and 6a–h was assessed against the MCF-7 cell line in comparison to Staurosporin (STU) as reference standard drug. Based on obtained *in vitro* results, the majority of the quinoline tethered *cis*-vinyl triamide derivatives tested demonstrated moderate to significant antiproliferative action against the examined cancer cell line. Five compounds; 5, 6b, 6d, 6e and 6f showed 1.06–7.10-fold more potent antiproliferative activity than the reference STU. Two compounds 6c and 6h showed antiproliferative activity with IC_50_ values of 19.25 and 15.63 μM, respectively comparable to STU (IC_50_ = 13.28 μM). The rest hybrids 6a and 6g showed modest antiproliferative activity with IC_50_ of 59.77 and 30.44 μM, respectively. The antiproliferative activity correlation of quinoline tethered *cis*-vinyl triamide derivatives 5 and 6a–h showed that among the quinoline-*cis* vinyl triamide derivatives, compounds bearing electron donating groups such as methyl; 6e (IC_50_ = 3.03 μM) and methoxy; 6f (IC_50_ = 1.87 μM) showed more potent antiproliferative activity than the other substituted derivatives with electron withdrawing groups and were found to be more potent than STU (IC_50_ = 13.28 μM). Another interesting observation is that introducing three groups in the 3,4,5-position of the phenyl ring attached to the *cis*-vinyl moiety, such as 3,5-dibromo-4-acetoxy group; 6g (IC_50_ = 30.44 μM) and 3,4,5-trimethoxy group; 6h (IC_50_ = 15.63 μM), resulted in low antiproliferative activity. It is also notable that replacement of substituted phenyl ring in *cis*-vinyl moiety with heterocyclic ring such as furan (IC_50_ = 6.60 μM) resulted in increase in antiproliferative activity compared to reference drug STU. According to these findings, the examined quinoline tethered triamide motif bearing *cis*-vinyl group with furan function or *para*-substituted phenyl ring with electron donating group such as methyl or methoxy had a beneficial influence on antiproliferative activity Table 1.

**Table 1:** *In vitro* cytotoxic results (IC_50_ values, μM) of quinoline tethered *cis*-vinyl triamides 5 and 6a–h*versus* examined cell line

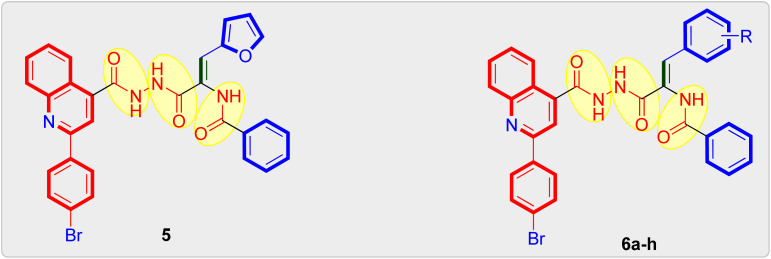		
Comp. no.	R	IC_50_ (μM)
		MCF-7
5	—	6.60 ± 0.21
6a	4-F	59.77 ± 1.93
6b	4-Cl	8.29 ± 0.27
6c	4-Br	19.25 ± 0.62
6d	4-NO_2_	12.43 ± 0.40
6e	4-CH_3_	3.03 ± 0.10
6f	4-OCH_3_	1.87 ± 0.06
6g	3,5-di-Br-4-OCOCH_3_	30.44 ± 0.98
6h	3,4,5-tri-OCH_3_	15.63 ± 0.42
STU	—	13.28 ± 0.43

#### 2.2.2. Cell cycle analysis

**Fig. 3:**
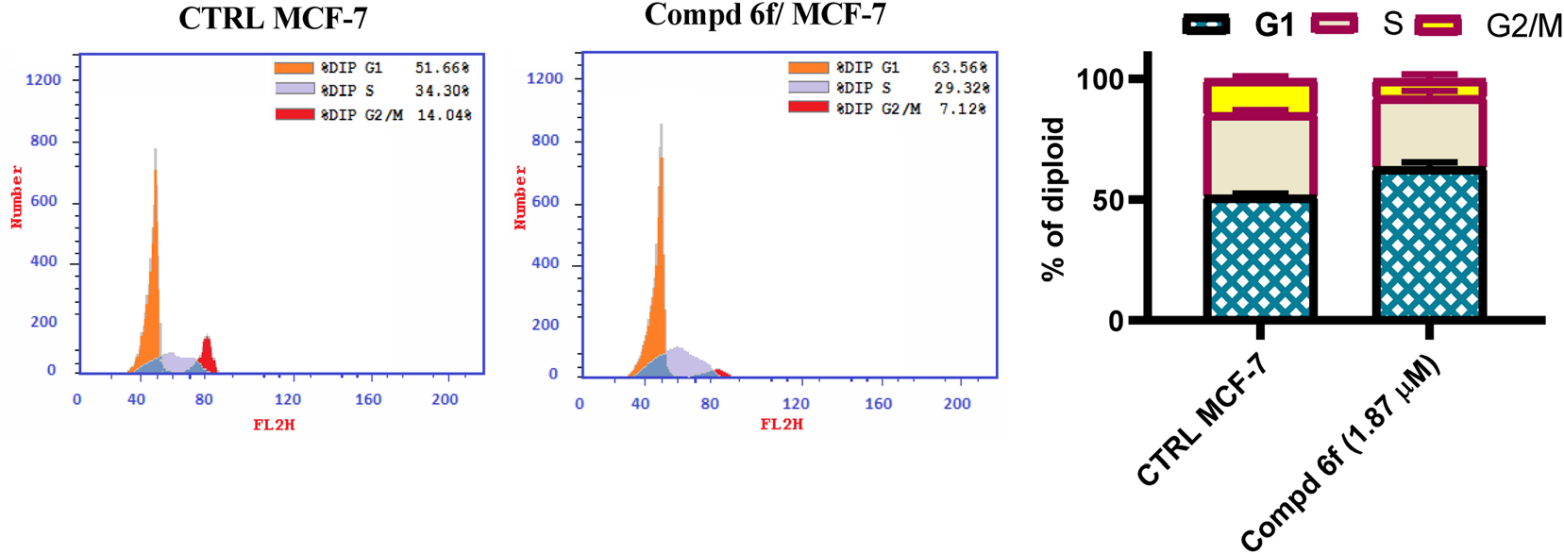
Sample graph of quinoline-[2-(4-methoxyphenyl)-*cis*-vinyl] triamide 6f treated MCF-7 cells compared to controls. This software calculates number of diploid cells in G1, S and G2 phase of cellular cycle.

The cell cycle is related to a series of events responsible for cell division and duplication.^42^ The cell cycle presents three distinct phases: G1, S and G2/M phase.^43^ Due to the importance of the cell cycle in the process of tumor progression, we evaluated if the cell growth inhibition in MCF-7 occurred due to cell cycle arrest using FACS method. In the present study, flow cytometric measurement was utilized to investigate the effect of quinoline tethered [2-(4-methoxyphenyl)-*cis*-vinyl] triamide 6f on cellular cycle progression in tested breast cancer cells. In the test, MCF-7 cells were treated with quinoline tethered [2-(4-methoxyphenyl)-*cis*-vinyl] triamide 6f at a concentration of 1.87 μM, then incubated for 48 h. The cellular cycle distribution was analyzed to identify the definite phase at which quinoline tethered [2-(4-methoxyphenyl)-*cis*-vinyl] triamide 6f can arrest the cell cycle. The results indicated that quinoline-[2-(4-methoxyphenyl)-*cis*-vinyl] triamide 6f significantly declined the cellular population at G1 phase. It is observed that the percentage of cells at G1 phase was increased by 1.2-fold compared to controls (Fig. 3). Coordinately, the percentage of cells was decreased at S and G2/M phases from 34.30 and 14.04% in controls to 29.32 and 7.12%, respectively in compound 6f-treated cells. These findings suggest that quinoline-[2-(4-methoxyphenyl)-*cis*-vinyl] triamide 6f halted the cell cycle proliferation of the MCF-7 cells at G1 phase.

#### 2.2.3. Apoptosis analysis

**Fig. 4:**
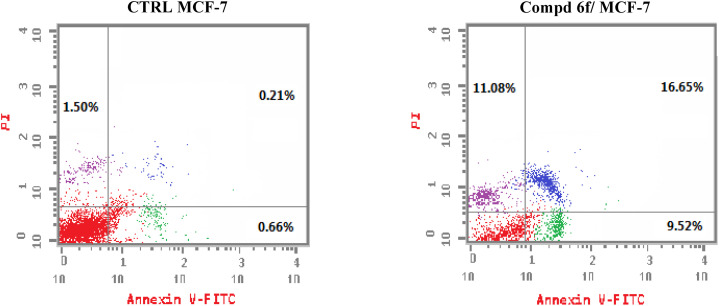
Sample graph showing the impact on apoptotic ratio caused by quinoline-[2-(4-methoxyphenyl)-*cis*-vinyl] triamide 6f-treated MCF-7 cells and compared to controls.

Apoptosis play an important role in organogenesis and in crafting complex tissues during embryonic growth, and in the preservation of tissue homeostasis in adult organism.^44^ Numerous clinical disorders, including cancer, autoimmune illness and infectious diseases, are characterised by the deregulation of apoptosis.^45^ To ensure the ability of quinoline tethered [2-(4-methoxyphenyl)-*cis*-vinyl] triamide 6f to activate apoptosis, flow cytometric measurement was carried out using Annexin V which binds to phophatidylserine declared on the outer layed of apoptotic cells and appears fluorescent green and PI which stains DNA and penetrates only dead cells. After 48 h of treatment with quinoline tethered [2-(4-methoxyphenyl)-*cis*-vinyl] triamide 6f at the IC_50_ concentration (1.87 μM), the percentage of cells that are survived was found to decrease. Additionally, there was a notable rise in the proportion of cells positive for Annexin V. It is notable that, the percentage of primary apoptotic cells was increased from 0.66% in DMSO controls to 9.52% in compound 6f-treated cells. Meanwhile, the proportion of late apoptotic cells rose from 0.21% to 16.65% compared to DMOS controls (Fig. 4). These findings suggest that quinoline tethered [2-(4-methoxyphenyl)-*cis*-vinyl] triamide 6f provoke apoptosis of MCF-7 cells.

#### 2.2.4. Mitochondrial membrane potential

**Fig. 5:**
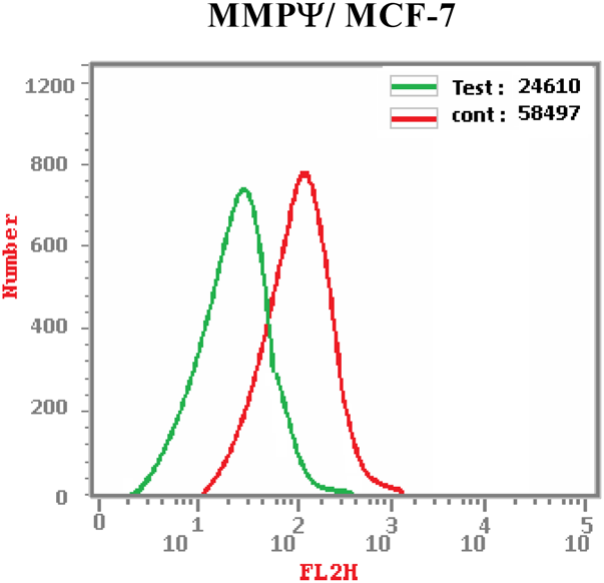
Sample graph showing the effect on MMP caused by quinoline-[2-(4-methoxyphenyl)-*cis*-vinyl] triamide 6f-treated MCF-7 cells and compared to controls.

A major part of apoptosis process is played by mitochondria.^46^ Several active proteins such as cytochrome C and factor that induces apoptosis are two of the active proteins found in mitochondria.^47^ The release of such proteins after inhibition of mitochondrial membrane potential (MMP) is crucial in the propagation of apoptosis.^48^ To confirm whether quinoline tethered [2-(4-methoxyphenyl)-*cis*-vinyl] triamide 6f could lower the MMP of tested cancer cell line (MCF-7 cells), flow cytometry was used to track MMP following treatment of the examined cells with 1.87 μM (IC_50_ dose value) of compound 6f. Results indicated that the tested cancer cells exhibited significant decrease in MMP. This decrease in MMP was linked to an increase in Annexin V positive cells. It is notable that, in comparison to DMSO controls, MCF-7 cells' MMP was 57.93% percent value lower (Fig. 5). According to this, quinoline tethered [2-(4-methoxyphenyl)-*cis*-vinyl] triamide 6f cause MCF-7 cells to become dysfunction in their mitochondria, which in turn trigger apoptotic cellular death. These outcomes were consistent with early findings that EGFR-TK inhibitors promote apoptosis *via* the mitochondrial route.

#### 2.2.5. EGFR tyrosine kinase inhibition analysis

**Fig. 6:**
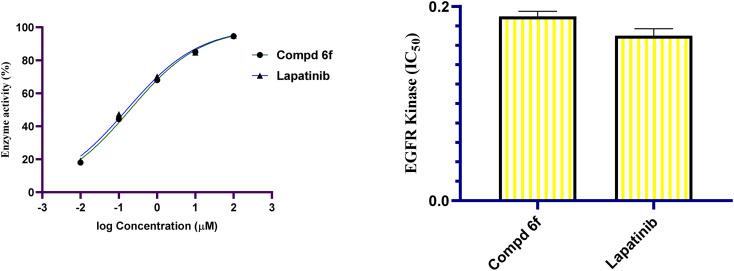
Graphical illustration of the EGFR inhibitory activity (IC_50_, μM) of quinoline-[2-(4-methoxyphenyl)-*cis*-vinyl] triamide 6f treated compared to Lapatinib.

EGFR have been implicated in a variety of cancer indications and other inappropriate mitogenic signalling disorders.^49^ Clinical evidence demonstrates an association between EGFR overexpression and response to a number of anticancer therapeutics.^50^ So EGFR and the activated downstream cascades represent a promising target for the establishment of therapeutic agents.^51^ As result of the effect of quinoline-[2-(4-methoxyphenyl)-*cis*-vinyl] triamide 6f on the G1 phase of the cellular cycle where the protein synthesis required for the cell growth rises, the inhibition of EGFR tyrosine kinase was thought to be the expected mode of cellular action of this hybrid molecule. Thus, the purpose of this experiment was to assess the inhibitory activity of EGFR tyrosine kinase. The quinoline-[2-(4-methoxyphenyl)-*cis*-vinyl] triamide 6f showed good activity (IC_50_ = 0.19 μM), which was comparable to that of Lapatinib (IC_50_ = 0.17 μM), a known potent EGFR inhibitor (Fig. 6). So it is concluded that quinoline-[2-(4-methoxyphenyl)-*cis*-vinyl] triamide 6f exerted its antiproliferative activity through inhibition of EGFR tyrosine kinase.

## 3. Conclusions

A new set of quinoline-tethered *cis*-vinyl triamide hybrids 5 and 6a–h was designed and constructed as inhibitors of EGFR tyrosine kinase for the treatment of breast cancer. All the prepared quinoline-tethered *cis*-vinyl triamide hybrids were screened for their *in vitro* antiproliferative activity and revealed moderate to potent activity. Among them, quinoline compounds 6e bearing [2-(4-methylphenyl)-*cis*-vinyl] triamide and 6f bearing [2-(4-methoxyphenyl)-*cis*-vinyl] triamide were found to be the most potent hybrids against the MCF-7 breast cancer cell line, with IC_50_ values of 1.87 and 1.88 μM, respectively, compared to STU (IC_50_ = 13.77 μM). Additional mechanistic studies demonstrated that quinoline-[2-(4-methoxyphenyl)-*cis*-vinyl] triamide 6f effectively blocked the G1 phase of the cell cycle and was found to promote cellular apoptosis. It is notable that quinoline-[2-(4-methoxyphenyl)-*cis*-vinyl] triamide 6f increased the percentage population at G1 phase by 1.2-fold more than controls. In addition, it boosted the level of both early and late apoptosis by almost 14.4- and 79.3-fold, respectively, compared to controls. Further mechanistic apoptotic studies confirmed that treatment in MCF-7 cells with the most active member; quinoline-[2-(4-methoxyphenyl)-*cis*-vinyl] triamide 6f, decreased the level of MMP of MCF-7 cells (57.93% percent change value) less than controls. Notably, quinoline-[2-(4-methoxyphenyl)-*cis*-vinyl] triamide 6f also demonstrated potent EGFR tyrosine kinase inhibition with an IC_50_ value of 0.19 μM, which was equipotent to reference standard Lapatinib (IC_50_ = 0.17 μM). The previous findings indicated that quinoline-[2-(4-methoxyphenyl)-*cis*-vinyl] triamide 6f is a potent apoptosis-active anticancer molecule that exerted its action *via* EGFR tyrosine kinase inhibition as a promising structure that could serve as a novel template for developing powerful and selective agents in cancer therapy.

## 4. Experimental

### 4.1. Chemistry

#### 4.1.1. General procedure for synthesis of (*Z*)-*N*-(1-aryl-3-(2-(2-(4-bromophenyl) quinoline-4-carbonyl)hydrazinyl)-3-oxoprop-1-en-2-yl)benzamides 5 and 6a–h

An appropriate *cis*-methyl 3-aryl-2-(benzamido)-2-propenoate (1 mmol) was added to the mixture of quinoline-4-carbohydrazide 4 (0.342 g, 1 mmol) and anhydrous sodium acetate (0.098 g, 1.2 mmol) in glacial acetic acid (20 mL) and refluxed for 20–22 h. After consumption of quinoline-4-carbohydrazide compound 4 (checked by TLC), the reaction mixture was cooled to ambient room temperature and poured onto cold water. The precipitated solid was separated by filtration, washed with cold water (15 mL) and dried. The crude solid was chromatographed on silica gel using ethyl acetate/*n*-hexane as eluent to attain target quinoline tethered *cis*-vinyl triamide 5 and 6a–h.

##### 4.1.1.1 (*Z*)-*N*-(3-(2-(2-(4-Bromophenyl)quinoline-4-carbonyl)hydrazinyl)-1-(furan-2-yl)-3-oxoprop-1-en-2-yl)benzamide (5)

Yield 76%; mp: 188–190 °C. ^1^H-NMR (400 MHz, DMSO-*d*_6_) *δ*: 10.80 (s, 1H, NH), 10.49 (s, 1H, NH), 9.99 (s, 1H, NH), 8.42 (d, *J* = 8.4 Hz, 1H, C8–H quinoline), 8.26 (d, *J* = 8.3 Hz, 2H), 8.19–8.14 (m, 2H), 8.13–8.07 (m, 2H), 7.88 (t, *J* = 7.6 Hz, C7–H quinoline), 7.84–7.78 (m, 3H), 7.71 (t, *J* = 7.7 Hz, 2H, C6–H quinoline and Ar–H), 7.63 (dd, *J* = 8.3, 6.2 Hz, 1H), 7.56 (t, *J* = 7.5 Hz, 2H), 7.35 (s, 1H), 6.82 (d, *J* = 3.5 Hz, 1H), 6.63 (dd, *J* = 3.5, 1.8 Hz, 1H). ^13^C-NMR (101 MHz, DMSO) *δ* 166.32, 166.11, 164.45, 155.01, 150.03, 148.30, 145.42, 141.99, 137.75, 134.30, 132.45, 132.14, 131.06, 129.97, 129.74, 128.75, 128.47, 127.98, 126.18, 126.01, 124.22, 124.05, 119.42, 117.14, 115.14, 112.88. C_30_H_21_BrN_4_O_4_ (581.42): calcd: C, 61.97; H, 3.64; N, 9.64. Found: C, 62.09; H, 3.78; N, 9.51.

##### 4.1.1.2. (*Z*)-*N*-(3-(2-(2-(4-Bromophenyl)quinoline-4-carbonyl)hydrazinyl)-1-(4-fluorophenyl)-3-oxoprop-1-en-2-yl)benzamide (6a)

Yield 68%; mp: 177–179 °C. ^1^H-NMR (400 MHz, DMSO-*d*_6_) *δ* 10.68 (d, *J* = 90.2 Hz, 2H, 2NH), 10.11 (s, 1H, NH), 8.46 (d, *J* = 8.4 Hz, 1H, C8–H quinoline), 8.31–8.23 (m, 2H, Ar–H), 8.16 (t, *J* = 4.3 Hz, 2H, Ar–H), 8.06 (d, *J* = 7.6 Hz, 2H, Ar–H), 7.87 (t, *J* = 7.6 Hz, 1H, C7–H quinoline), 7.83–7.77 (m, 2H, Ar–H), 7.71 (td, *J* = 5.6, 2.9 Hz, 3H, C6–H quinoline and Ar–H), 7.65–7.59 (m, 1H, Ar–H), 7.55 (t, *J* = 7.4 Hz, 2H, Ar–H), 7.43 (s, 1H, =CH), 7.26 (td, *J* = 8.9, 1.6 Hz, 2H, Ar–H). ^13^C-NMR (101 MHz, DMSO) *δ* 168.85, 166.50, 166.05, 156.50, 155.00, 148.32, 142.34, 142.19, 137.80, 134.03, 132.45, 132.25, 132.21, 132.13, 131.06, 131.00, 129.96, 129.74, 128.79, 128.47, 127.91, 126.12, 124.22, 117.14, 116.19, 115.98. C_32_H_22_BrFN_4_O_3_ (609.44): calcd: C, 63.06; H, 3.64; N, 9.19. Found: C, 62.95; H, 3.55; N, 9.33.

##### 4.1.1.3. (*Z*)-*N*-(3-(2-(2-(4-Bromophenyl)quinoline-4-carbonyl)hydrazinyl)-1-(4-chlorophenyl)-3-oxoprop-1-en-2-yl)benzamide (6b)

Yield 73%; mp: 185–187 °C. ^1^H-NMR (400 MHz, DMSO-*d*_6_) *δ* 10.83 (s, 1H, NH), 10.58 (s, 1H, NH), 10.13 (s, 1H, NH), 8.43 (d, *J* = 8.4 Hz, 1H, C8–H quinoline), 8.27 (d, *J* = 8.2 Hz, 2H, Ar–H), 8.16 (s, 2H, Ar–H), 8.08–8.02 (m, 2H, Ar–H), 7.88 (t, *J* = 7.6 Hz, 1H, C7–H quinoline), 7.81 (d, *J* = 8.2 Hz, 2H, Ar–H), 7.72 (t, *J* = 7.7 Hz, 2H, C6–H quinoline), 7.67 (d, *J* = 8.3 Hz, 2H, Ar–H), 7.62 (d, *J* = 7.2 Hz, 1H, Ar–H), 7.55 (t, *J* = 7.5 Hz, 2H, Ar–H), 7.48 (d, *J* = 8.3 Hz, 2H, Ar–H), 7.40 (s, 1H, =CH). ^13^C-NMR (101 MHz, DMSO) *δ* 166.49, 166.15, 164.96, 155.02, 148.30, 141.98, 137.74, 133.95, 133.84, 133.43, 132.46, 132.29, 131.61, 131.07, 129.98, 129.80, 129.75, 129.53, 129.13, 128.79, 128.48, 128.00, 125.98, 124.23, 124.05, 117.15. C_32_H_22_BrClN_4_O_3_ (625.90): calcd: C, 61.41; H, 3.54; N, 8.95. Found: C, 61.59; H, 3.39; N, 9.09.

##### 4.1.1.4. (*Z*)-*N*-(1-(4-Bromophenyl)-3-(2-(2-(4-bromophenyl)quinoline-4-carbonyl)hydrazinyl)-3-oxoprop-1-en-2-yl)benzamide (6c)

Yield 78%; mp: 191–193 °C. ^1^H-NMR (400 MHz, DMSO-*d*_6_) *δ* 10.83 (s, 1H, NH), 10.58 (s, 1H, NH), 10.13 (s, 1H, NH), 8.43 (d, *J* = 8.4 Hz, 1H, C8–H quinoline), 8.26 (d, *J* = 8.2 Hz, 2H, Ar–H), 8.17 (d, *J* = 7.7 Hz, 2H, Ar–H), 8.05 (d, *J* = 7.7 Hz, 2H, Ar–H), 7.87 (t, *J* = 7.8 Hz, 1H, C7–H quinoline), 7.80 (d, *J* = 8.1 Hz, 2H, Ar–H), 7.71 (t, *J* = 7.7 Hz, 2H, C6–H quinoline), 7.60 (h, *J* = 8.0, 7.3 Hz, 6H, Ar–H), 7.37 (s, 1H, =CH). ^13^C-NMR (101 MHz, DMSO) *δ* 166.46, 166.14, 164.95, 155.02, 148.31, 141.99, 137.75, 133.96, 133.80, 132.46, 132.29, 132.05, 131.84, 131.07, 129.99, 129.91, 129.75, 129.55, 128.79, 128.49, 127.99, 126.00, 124.23, 124.06, 122.59, 117.15. C_32_H_22_Br_2_N_4_O_3_ (670.35): calcd: C, 57.33; H, 3.31; N, 8.36. Found: C, 57.42; H, 3.36; N, 8.29.

##### 4.1.1.5. (*Z*)-*N*-(3-(2-(2-(4-Bromophenyl)quinoline-4-carbonyl)hydrazinyl)-1-(4-nitrophenyl)-3-oxoprop-1-en-2-yl)benzamide (6d)

Yield 69%; mp: 207–209 °C. ^1^H-NMR (400 MHz, DMSO-*d*_6_) *δ* 10.89 (s, 1H, NH), 10.72 (s, 1H, NH), 10.30 (s, 1H, NH), 8.44 (d, *J* = 8.3 Hz, 1H, C8–H quinoline), 8.27 (dt, *J* = 9.0, 2.9 Hz, 4H, Ar–H), 8.18 (t, *J* = 4.2 Hz, 2H, Ar–H), 8.05 (d, *J* = 7.7 Hz, 2H, Ar–H), 7.90 (d, *J* = 8.7 Hz, 3H, Ar–H), 7.81 (d, *J* = 8.1 Hz, 2H, Ar–H), 7.72 (t, *J* = 7.7 Hz, 1H, C7–H quinoline), 7.63 (t, *J* = 7.4 Hz, 1H, C6–H quinoline), 7.56 (t, *J* = 7.5 Hz, 2H, Ar–H), 7.43 (s, 1H, =CH). ^13^C-NMR (101 MHz, DMSO) *δ* 166.53, 166.14, 164.73, 155.03, 148.31, 147.25, 142.33, 141.88, 141.54, 137.74, 132.45, 132.37, 131.07, 130.83, 129.99, 129.76, 128.82, 128.55, 128.01, 127.79, 127.62, 125.96, 125.90, 124.17, 124.05, 117.18. C_32_H_22_BrN_5_O_5_ (636.45): calcd: C, 60.39; H, 3.48, N; 11.00. Found: C, 60.24; H, 3.57; N, 11.12.

##### 4.1.1.6. (*Z*)-*N*-(3-(2-(2-(4-Bromophenyl)quinoline-4-carbonyl)hydrazinyl)-3-oxo-1-*p*-tolylprop-1-en-2-yl)benzamide (6e)

Yield 70%; mp: 211–213 °C. ^1^H-NMR (400 MHz, DMSO-*d*_6_) *δ* 10.79 (s, 1H, NH), 10.49 (s, 1H, NH), 10.07 (s, 1H, NH), 8.44 (d, *J* = 8.4 Hz, 1H, C8–H quinoline), 8.27 (d, *J* = 8.3 Hz, 2H, Ar–H), 8.17 (d, *J* = 9.0 Hz, 2H, Ar–H), 8.07 (d, *J* = 7.6 Hz, 2H, Ar–H), 7.88 (t, *J* = 7.5 Hz, 1H, C7–H quinoline), 7.81 (d, *J* = 8.2 Hz, 2H, Ar–H), 7.72 (t, *J* = 7.8 Hz, 1H, C6–H quinoline), 7.58 (dt, *J* = 15.1, 7.2 Hz, 5H, Ar–H), 7.42 (s, 1H, =CH), 7.22 (d, *J* = 7.9 Hz, 2H, Ar–H), 2.32 (s, 3H, Ar–CH_3_). ^13^C-NMR (101 MHz, DMSO) *δ* 167.67, 166.78, 166.10, 164.42, 155.03, 148.30, 145.77, 141.91, 137.74, 135.63, 133.91, 133.45, 132.42, 131.43, 131.08, 130.00, 129.75, 128.82, 128.38, 128.02, 127.40, 125.96, 124.24, 124.04, 117.64, 117.15, 20.65. C_33_H_25_BrN_4_O_3_ (605.48): calcd: C, 65.46; H, 4.16; N, 9.25. Found: C, 65.60; H, 4.28; N, 9.11.

##### 4.1.1.7. (*Z*)-*N*-(3-(2-(2-(4-Bromophenyl)quinoline-4-carbonyl)hydrazinyl)-1-(4-methoxyphenyl)-3-oxoprop-1-en-2-yl)benzamide (6f)

Yield 75%; mp: 206–208 °C. ^1^H-NMR (400 MHz, DMSO-*d*_6_) *δ* 10.76 (s, 1H, NH), 10.43 (s, 1H, NH), 10.04 (s, 1H, NH), 8.44 (d, *J* = 8.4 Hz, 1H, C8–H quinoline), 8.30–8.23 (m, 2H, Ar–H), 8.19–8.13 (m, 2H, Ar–H), 8.12–8.05 (m, 2H, Ar–H), 7.87 (t, *J* = 7.5 Hz, 1H, C7–H quinoline), 7.84–7.77 (m, 2H, Ar–H), 7.71 (t, *J* = 7.8 Hz, 1H, C6–H quinoline), 7.63 (d, *J* = 8.5 Hz, 3H, Ar–H), 7.55 (t, *J* = 7.4 Hz, 2H, Ar–H), 7.43 (s, 1H, =CH), 6.98 (d, *J* = 8.8 Hz, 2H, Ar–H), 3.78 (s, 3H, Ar–OCH_3_). ^13^C-NMR (101 MHz, DMSO) *δ* 166.44, 166.16, 165.27, 160.36, 155.01, 142.10, 140.27, 137.76, 134.18, 132.46, 132.17, 131.83, 131.42, 130.83, 129.97, 129.74, 128.77, 128.46, 127.97, 126.85, 126.75, 126.04, 124.22, 124.07, 117.13, 114.60, 55.72. C_33_H_25_BrN_4_O_4_ (621.48): calcd; C, 63.78; H, 4.05; N, 9.02. Found: C, 63.86; H, 3.96; N, 8.95.

##### 4.1.1.8. (*Z*)-4-(2-Benzamido-3-(2-(2-(4-bromophenyl)quinoline-4-carbonyl)hydrazinyl)-3-oxoprop-1-enyl)-2,6-dibromophenyl acetate (6g)

Yield 64%; mp: 181–183 °C. ^1^H-NMR (400 MHz, DMSO-*d*_6_) *δ* 10.86 (s, 1H, NH), 10.65 (s, 1H, NH), 10.22 (s, 1H, NH), 8.43 (d, *J* = 8.5 Hz, 1H, C8–H quinoline), 8.27 (d, *J* = 8.4 Hz, 2H, Ar–H), 8.17 (d, *J* = 8.8 Hz, 2H, Ar–H), 8.03 (d, *J* = 10.7 Hz, 4H, Ar–H), 7.88 (t, *J* = 7.6 Hz, C7–H quinoline), 7.81 (d, *J* = 8.3 Hz, 2H, Ar–H), 7.71 (t, *J* = 7.8 Hz, 1H, C6–H quinoline), 7.63 (t, *J* = 7.2 Hz, 1H, Ar–H), 7.56 (t, *J* = 7.5 Hz, 2H, Ar–H), 7.40 (s, 1H, =CH), 2.40 (s, 3H, Ar–OCOCH_3_). ^13^C-NMR (101 MHz, DMSO) *δ* 166.48, 166.18, 165.19, 165.17, 155.02, 148.31, 142.06, 139.30, 137.76, 134.15, 132.47, 132.20, 131.61, 131.39, 131.08, 130.04, 129.75, 129.69, 128.77, 128.45, 128.23, 128.00, 126.05, 126.01, 124.24, 124.07, 117.13, 21.41. C_34_H_23_Br_3_N_4_O_5_ (807.28): calcd: C, 50.59; H, 2.87; N, 6.94. Found: C, 50.44; H, 2.79; N, 7.07.

##### 4.1.1.9. (Z)-*N*-(3-(2-(2-(4-Bromophenyl)quinoline-4-carbonyl)hydrazinyl)-3-oxo-1-(3,4,5-trimethoxyphenyl)prop-1-en-2-yl)benzamide (6h)

Yield 67%; mp: 169–171 °C. ^1^H-NMR (400 MHz, DMSO-*d*_6_) *δ* 10.80 (s, 1H, NH), 10.51 (s, 1H, NH), 10.10 (s, 1H, NH), 8.45 (dd, *J* = 8.4, 1.4 Hz, 1H, C8–H quinoline), 8.31–8.23 (m, 2H, Ar–H), 8.17 (d, *J* = 10.0 Hz, 2H, Ar–H), 8.12 (dd, *J* = 7.1, 2.0 Hz, 2H, Ar–H), 7.88 (t, *J* = 7.5 Hz, C7–H quinoline), 7.84–7.78 (m, 2H, Ar–H), 7.72 (t, *J* = 7.7 Hz, 1H, C6–H quinoline), 7.62 (t, *J* = 7.4 Hz, 1H, Ar–H), 7.55 (t, *J* = 7.5 Hz, 2H, Ar–H), 7.48 (s, 1H, =CH), 7.03 (s, 2H, Ar–H), 3.68 (s, 3H, OCH_3_), 3.63 (s, 6H, 2OCH_3_). ^13^C-NMR (101 MHz, DMSO) *δ* 166.48, 166.13, 164.95, 155.02, 153.09, 152.91, 148.31, 142.04, 138.72, 137.76, 134.00, 132.46, 132.28, 131.90, 131.07, 129.98, 129.74, 128.71, 128.40, 128.14, 127.97, 126.03, 124.23, 124.07, 117.13, 107.71, 60.54, 56.08. C_35_H_29_BrN_4_O_6_ (681.53): calcd: C, 61.68; H, 4.29; N, 8.22. Found: C, 61.81; H, 4.36; N, 8.10.

### 4.2. Biological studies

All the experimental procedure used in the biological studies were shown in the ESI.†

## Data availability

The authors confirm that the data supporting the findings of this study are available within the article and/or its ESI.†

## Conflicts of interest

No potential conflict of interest was reported by the author(s).

## Supplementary Material

RA-014-D4RA04915B-s001

## References

[cit1] Santucci C., Mignozzi S., Malvezzi M., Boffetta P., Collatuzzo G., Levi F., La Vecchia C., Negri E. (2024). European cancer mortality predictions for the year 2024 with focus on colorectal cancer. Ann. Oncol..

[cit2] Siegel R. L., Giaquinto A. N., Jemal A. (2024). Cancer statistics. Ca-Cancer J. Clin..

[cit3] Nenclares P., Harrington K. J. (2020). The biology of cancer. Medicine.

[cit4] Cuthrell K. M., Tzenios N. (2023). Breast Cancer: Updated and Deep Insights. Int. Res. J. Oncol..

[cit5] Ensenyat-Mendez M., Llinàs-Arias P., Orozco J. I. J., Íñiguez-Muñoz S., Salomon M. P., Sesé B., DiNome M. L., Marzese D. M. (2021). Current Triple-Negative Breast Cancer Subtypes: Dissecting the Most Aggressive Form of Breast Cancer. Front. Oncol..

[cit6] Wang J., Wu S.-G. (2023). Breast Cancer: An Overview of Current Therapeutic Strategies, Challenge, and Perspectives. Breast Cancer: Targets Ther..

[cit7] Elmore J. G., Lee C. I. (2024). Toward More Equitable Breast Cancer Outcomes. JAMA.

[cit8] Brianna, Lee S. H. (2023). Chemotherapy: how to reduce its adverse effects while maintaining the potency?. Med. Oncol..

[cit9] Puzzo M., De Santo M., Morelli C., Leggio A., Pasqua L. (2024). The Advent of Molecular Targeted Therapies Against Cancer. Toward Multi-Targeting Drugs Through Materials Engineering: A Possible Future Scenario. Small Sci..

[cit10] Jin H., Wang L., Bernards R. (2023). Rational combinations of targeted cancer therapies: background, advances and challenges. Nat. Rev. Drug Discovery.

[cit11] Uribe M. L., Marrocco I., Yarden Y. (2021). EGFR in Cancer: Signaling Mechanisms, Drugs, and Acquired Resistance. Cancers.

[cit12] Burgess A. W. (2022). Regulation of Signaling from the Epidermal Growth Factor Family. J. Phys. Chem. B.

[cit13] Dang A., Dang S., Vallish B. (2021). Efficacy and Safety of EGFR Inhibitors in the Treatment of EGFRPositive NSCLC Patients: A Meta-analysis. Rev. Recent Clin. Trials.

[cit14] Liu S.-Y., Liu S.-Y. M., Zhong W.-Z., Wu Y.-L. (2022). Targeted Therapy in Early Stage Non-small Cell Lung Cancer. Curr. Treat. Options Oncol..

[cit15] Halder S., Basu S., Lall S. P., Ganti A. K., Batra S. K., Seshacharyulu P. (2023). Targeting the EGFR signaling pathway in cancer therapy: What's new in 2023?. Expert Opin. Ther. Targets.

[cit16] Alharbi K. S., Javed Shaikh M. A., Afzal O., Alfawaz Altamimi A. S., Almalki W. H., Alzarea S. I., Kazmi I., Al-Abbasi F. A., Singh S. K., Dua K., Gupta G. (2022). An overview of epithelial growth factor receptor (EGFR) inhibitors in cancer therapy. Chem. Biol. Interact..

[cit17] Volta F., La Monica S., Leonetti A., Gnetti L., Bonelli M., Cavazzoni A., Fumarola C., Galetti M., Eltayeb K., Minari R., Petronini P. G., Tiseo M., Alfieri R. (2023). Intrinsic Resistance to Osimertinib in EGFR Mutated NSCLC Cell Lines Induced by Alteration in Cell-Cycle Regulators. Targeted Oncol..

[cit18] Nasser Binjawhar D., Al-Salmi F. A., Alghamdi M. A., Alqahtani A. s., Fayad E., Saleem R. M., Zaki I., Youssef Moustafa A. M. (2024). Design, Synthesis, and Biological Evaluation of Newly Synthesized Cinnamide-Fluorinated Containing Compounds as Bioactive Anticancer Agents. ACS Omega.

[cit19] Roskoski R. (2024). Properties of FDA-approved small molecule protein kinase inhibitors: A 2024 update. Pharmacol. Res..

[cit20] Ryad N., Elmaaty A. A., M Ibrahim I., Ahmed Maghrabi A. H., Yahya Alahdal M. A., Saleem R. M., Zaki I., Ghany L. M. A. A. (2024). Harnessing molecular hybridization approach to discover novel quinoline EGFR-TK inhibitors for cancer treatment. Future Med. Chem..

[cit21] Yadav V., Reang J., Sharma V., Majeed J., Sharma P. C., Sharma K., Giri N., Kumar A., Tonk R. K. (2022). Quinoline-derivatives as privileged scaffolds for medicinal and pharmaceutical chemists: A comprehensive review. Chem. Biol. Drug Des..

[cit22] Ajani O. O., Iyaye K. T., Ademosun O. T. (2022). Recent advances in chemistry and therapeutic potential of functionalized quinoline motifs–a review. RSC Adv..

[cit23] Binjawhar D. N., Al-Salmi F. A., Ali O. A. A., Alghamdi M. A., Fayad E., Saleem R. M., Zaki I., Farouk N. (2024). Design, synthesis and cytotoxic activity of molecular hybrids based on quinolin-8-yloxy and cinnamide hybrids and their apoptosis inducing property. RSC Adv..

[cit24] dos Santos Chagas C., Fonseca F. L. A., Bagatin I. A. (2019). Quinoline-derivative coordination compounds as potential applications to antibacterial and antineoplasic drugs. Mater. Sci. Eng. C.

[cit25] Mohasin M., Zafer Alam M., Ullah Q., Ahmad A., Rahaman P. F., Khan S. A. (2024). A Review on Synthesis and Biological Applications of Quinoline Derivative as Fused Aromatic Compounds. Polycyclic Aromat. Compd..

[cit26] Ilakiyalakshmi M., Arumugam Napoleon A. (2022). Review on recent development of quinoline for anticancer activities. Arabian J. Chem..

[cit27] Hu S., Chen J., Cao J.-X., Zhang S.-S., Gu S.-X., Chen F.-E. (2023). Quinolines and isoquinolines as HIV-1 inhibitors: Chemical structures, action targets, and biological activities. Bioorg. Chem..

[cit28] Abd El-Lateef H. M., Elmaaty A. A., Abdel Ghany L. M. A., Abdel-Aziz M. S., Zaki I., Ryad N. (2023). Design and Synthesis of 2-(4-Bromophenyl)Quinoline-4-Carbohydrazide Derivatives via Molecular Hybridization as Novel Microbial DNA-Gyrase Inhibitors. ACS Omega.

[cit29] Bala I. A., Al Sharif O. F., Asiri A. M., El-Shishtawy R. M. (2024). Quinoline: A versatile bioactive scaffold and its molecular hybridization. Results Chem..

[cit30] Snehi V., Verma H., Saha S., Kumar S., Pathak D. (2023). An extensive review on biological interest of quinoline and its analogues. Int. J. Sci. Healthcare Res..

[cit31] Chauhan Y., Neha K., Wakode S., Shahfaiz M., Bodla R. B., Sharma K. (2023). Progression and Expansion of Quinoline as Bioactive Moiety: A Patent Review. Pharm. Pat. Anal..

[cit32] Ren Y., Ruan Y., Cheng B., Li L., Liu J., Fang Y., Chen J. (2021). Design, synthesis and biological evaluation of novel acridine and quinoline derivatives as tubulin polymerization inhibitors with anticancer activities. Bioorg. Med. Chem..

[cit33] Swedan H. K., Kassab A. E., Gedawy E. M., Elmeligie S. E. (2023). Topoisomerase II inhibitors design: Early studies and new perspectives. Bioorg. Chem..

[cit34] Fan T., Ji Y., Chen D., Peng X., Ai J., Xiong B. (2023). Design, synthesis and biological evaluation of 4-aminoquinoline derivatives as receptor-interacting protein kinase 2 (RIPK2) inhibitors. J. Enzyme Inhib. Med. Chem..

[cit35] Hamdy R., Elseginy S. A., Ziedan N. I., Jones A. T., Westwell A. D. (2019). New Quinoline-Based Heterocycles as Anticancer Agents Targeting Bcl-2. Molecules.

[cit36] Abd El-Lateef H. M., Toson E. E. M., Abu Almaaty A. H., Saleem R. M., Maghrabi A. H. A., El-Sayed E. H., Zaki I., Youssef M. M. (2023). Synthesis, Characterization and Biological Evaluation of New Enamide Fluorinated-Schiff Base Derivatives as Potential Cytotoxic and Apoptosis-Inducing Agents. ChemistrySelect.

[cit37] Zhao C., Wang Y., Pham Q., Dai C., Chatterjee A., Wasa M. (2023). Chemical Tagging of Bioactive Amides by Cooperative Catalysis: Applications in the Syntheses of Drug Conjugates. J. Am. Chem. Soc..

[cit38] El-Fakharany Z. S., Nissan Y. M., Sedky N. K., Arafa R. K., Abou-Seri S. M. (2023). New proapoptotic chemotherapeutic agents based on the quinolone-3-carboxamide scaffold acting by VEGFR-2 inhibition. Sci. Rep..

[cit39] Mohamed K. O., Zaki I., El-Deen I. M., Abdelhameid M. K. (2019). A new class of diamide scaffold: Design, synthesis and biological evaluation as potent antimitotic agents, tubulin polymerization inhibition and apoptosis inducing activity studies. Bioorg. Chem..

[cit40] Abd El-Lateef H. M., Ghany L. M. A., Saleem R. M., Maghrabi A. H. A., Alahdal M. A. Y., Ali E. H. K., Beshay B. Y., Zaki I., Masoud R. E. (2023). Design, synthesis and antiproliferative screening of newly synthesized coumarin-acrylamide hybrids as potential cytotoxic and apoptosis inducing agents. RSC Adv..

[cit41] Ghany L. M. A. A., Ryad N., Abdel-Aziz M. S., El-Lateef H. M. A., Zaki I., Beshay B. Y. (2024). Design, synthesis, antimicrobial evaluation, and molecular modeling of new sulfamethoxazole and trimethoprim analogs as potential DHPS/DHFR inhibitors. J. Mol. Struct..

[cit42] Gousias K., Theocharous T., Simon M. (2022). Mechanisms of Cell Cycle Arrest and Apoptosis in Glioblastoma. Biomed.

[cit43] Guo X., Chen L. (2024). From G1 to M: a comparative study of methods for identifying cell cycle phases. Briefings Bioinf..

[cit44] Bertheloot D., Latz E., Franklin B. S. (2021). Necroptosis, pyroptosis and apoptosis: an intricate game of cell death. Cell. Mol. Immunol..

[cit45] Ozyerli-Goknar E., Bagci-Onder T. (2021). Epigenetic Deregulation of Apoptosis in Cancers. Cancers.

[cit46] Dadsena S., Zollo C., García-Sáez A. J. (2021). Mechanisms of mitochondrial cell death. Biochem. Soc. Trans..

[cit47] Abate M., Festa A., Falco M., Lombardi A., Luce A., Grimaldi A., Zappavigna S., Sperlongano P., Irace C., Caraglia M., Misso G. (2020). Mitochondria as playmakers of apoptosis, autophagy and senescence. Semin. Cell Dev. Biol..

[cit48] Takács-Vellai K. (2023). Apoptosis and Autophagy, Different Modes of Cell Death: How to Utilize Them to Fight Diseases?. Int. J. Mol. Sci..

[cit49] Liu H., Zhang B., Sun Z. (2020). Spectrum of EGFR aberrations and potential clinical implications: insights from integrative pan-cancer analysis. Cancer Commun..

[cit50] Alsahafi E. N., Thavaraj S., Sarvestani N., Novoplansky O., Elkabets M., Ayaz B., Tavassoli M., Legends M. F. (2021). EGFR overexpression increases radiotherapy response in HPV-positive head
and neck cancer through inhibition of DNA damage repair and HPV E6 downregulation. Cancer Lett..

[cit51] Maennling A. E., Tur M. K., Niebert M., Klockenbring T., Zeppernick F., Gattenlöhner S., Meinhold-Heerlein I., Hussain A. F. (2019). Molecular Targeting Therapy against EGFR Family in Breast Cancer: Progress and Future Potentials. Cancers.

