# Supplementary material for: Design, synthesis, and antiproliferative screening of new quinoline derivatives bearing a cis-vinyl triamide motif as apoptosis activators and EGFR-TK inhibitors
Source: RSC Adv. 2024 Aug 7;14(34):24781–90. doi: 10.1039/d4ra04915b (PMC11305403; doi:10.1039/d4ra04915b)
Supplement: RA-014-D4RA04915B-s001 [file RA-014-D4RA04915B-s001.pdf]

Supplementary Information (SI) for RSC Advances.
This journal is © The Royal Society of Chemistry 2024

Hany M. Abd El-Lateef [1,2,*], Ahmed Gaafar A. Gaafar [3], Arwa Sultan Alqahtani [4], Amal A. Al-Mutairi [4], Dalal Sulaiman Alshaya [5], Fahmy Gad Elsaid [6], Eman Fayad [7], N. A. Farouk [8,*]

[1] Department of Chemistry, College of Science, King Faisal University, Al-Ahsa 31982, Saudi Arabia

[2] Department of Chemistry, Faculty of Science, Sohag University, Sohag 82524, Egypt, hmahmed@kfu.edu.sa

[3] Department of Pharmacology and Toxicology, Faculty of Pharmacy, Port Said University, Port Said, Egypt

[4] Department of Chemistry, College of Science, Imam Mohammad Ibn Saud Islamic University(IMSIU), P.O. Box, 90950, Riyadh 11623, Saudi Arabia

[5] Department of Biology, College of Science, Princess Nourah bint Abdulrahman University, P.O. Box 84428, Riyadh 11671, Saudi Arabia

[6] Department of Biology, College of Science, King Khalid University, PO Box 960, Asir, Abha, 61421, Saudi Arabia

[7] Department of Biotechnology, College of Sciences, Taif University, P.O. Box 11099, Taif 21944, Saudi Arabia

[8] Department of Chemistry, Faculty of Science, Port Said University, Port Said, Egypt

---

* *To whome correspondence should be addressed*

N. A. Farouk, PhD. Department of Chemistry, Faculty of Science, Port Said University, Port Said, Egypt

E-mail address: nesmafarouk566@gmail.com **(Farouk N. A.)**

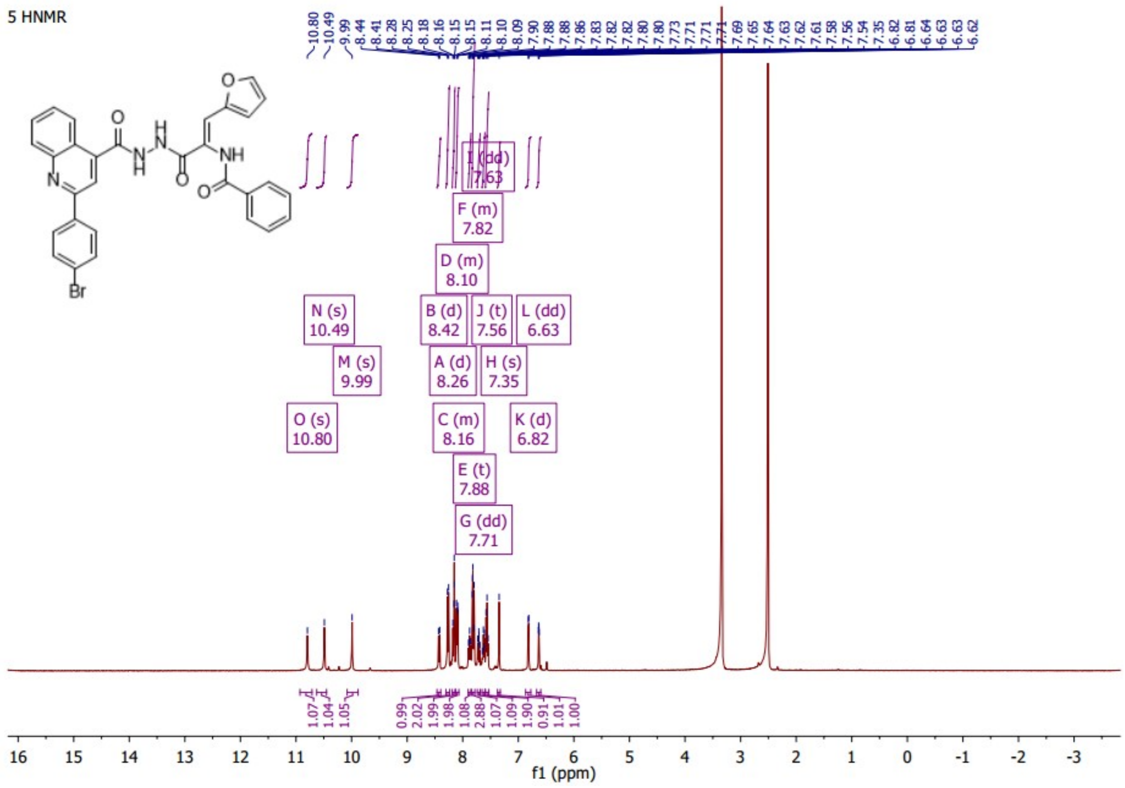

Figure S1: ^{1}H-NMR spectrum of compound **5**

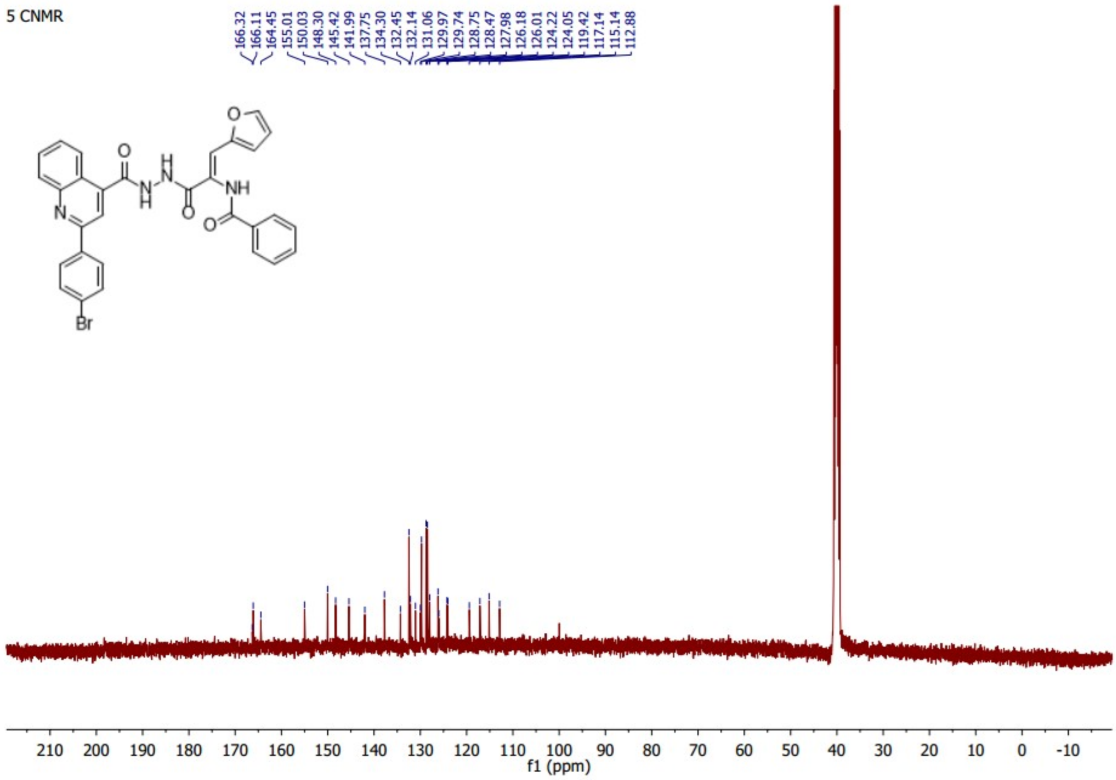

Figure S2: ^{13}C-NMR spectrum of compound **5**

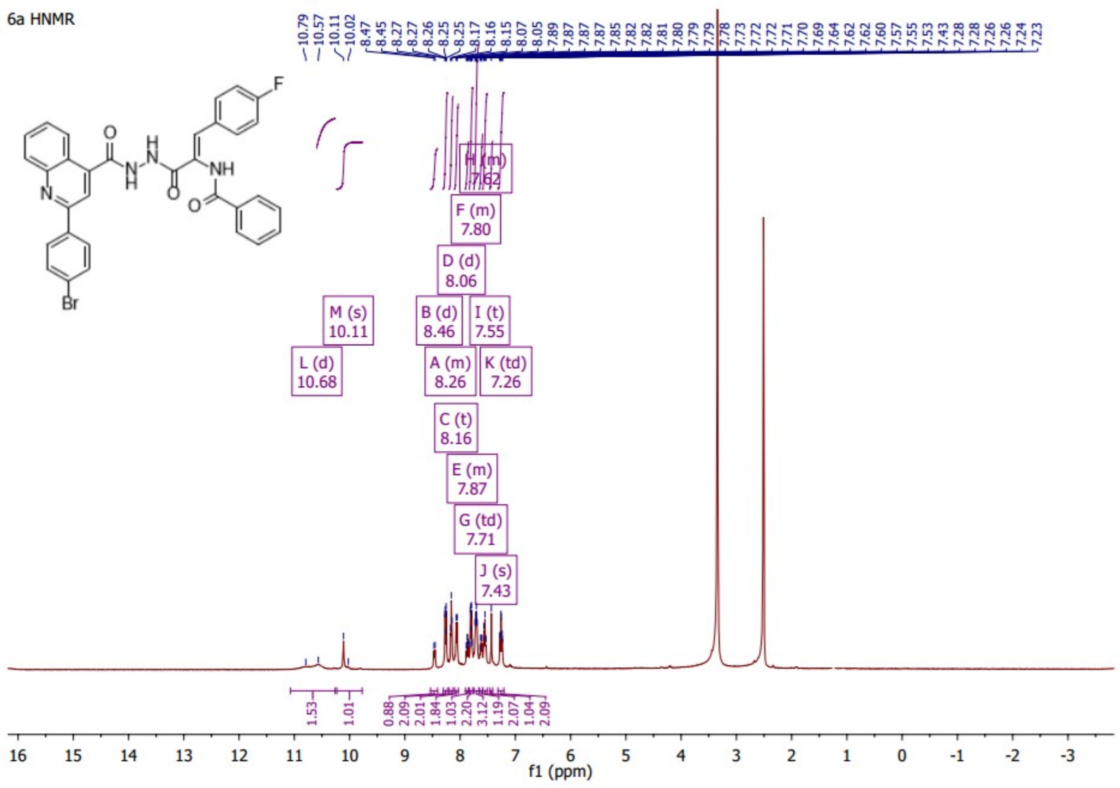

Figure S3: ^{1}H-NMR spectrum of compound **6a**

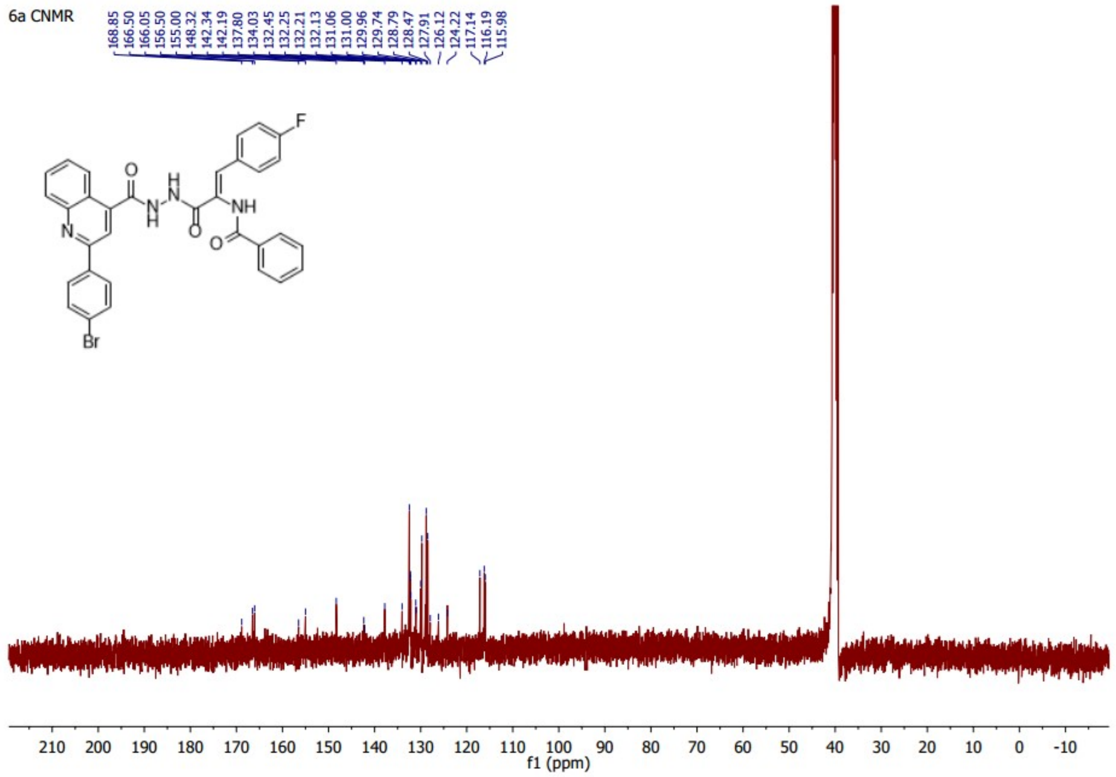

Figure S4: ^{13}C-NMR spectrum of compound **6a**

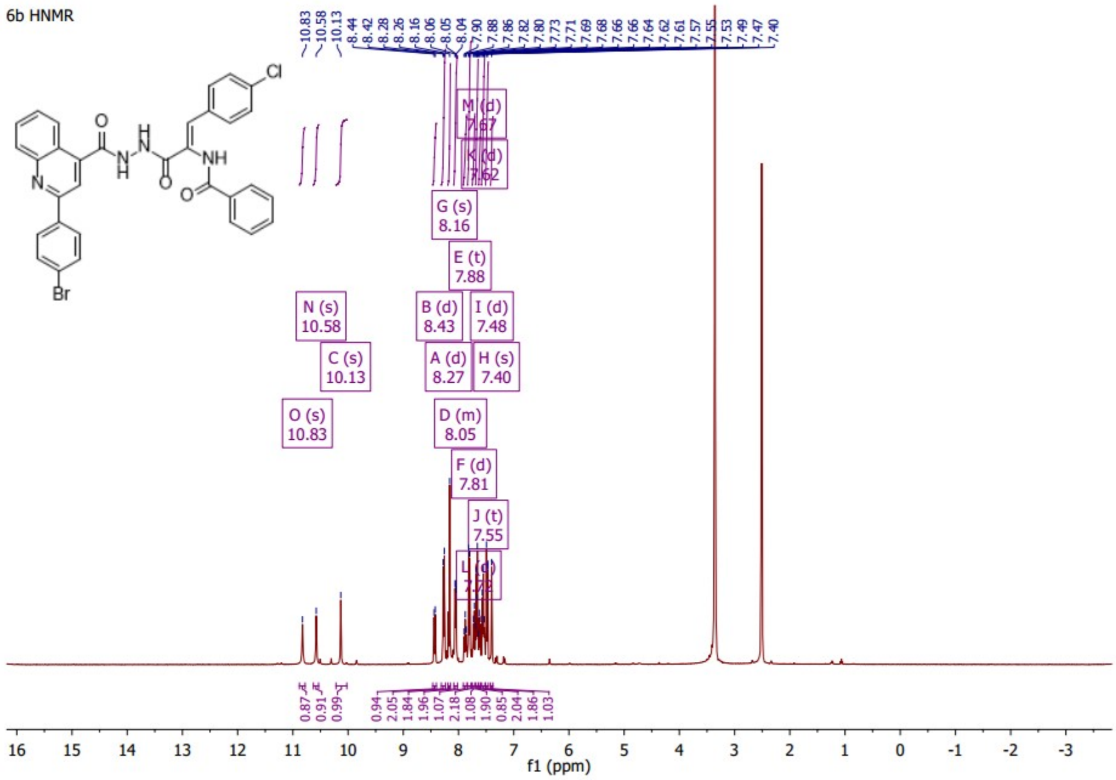

Figure S5: ^{1}H-NMR spectrum of compound **6b**

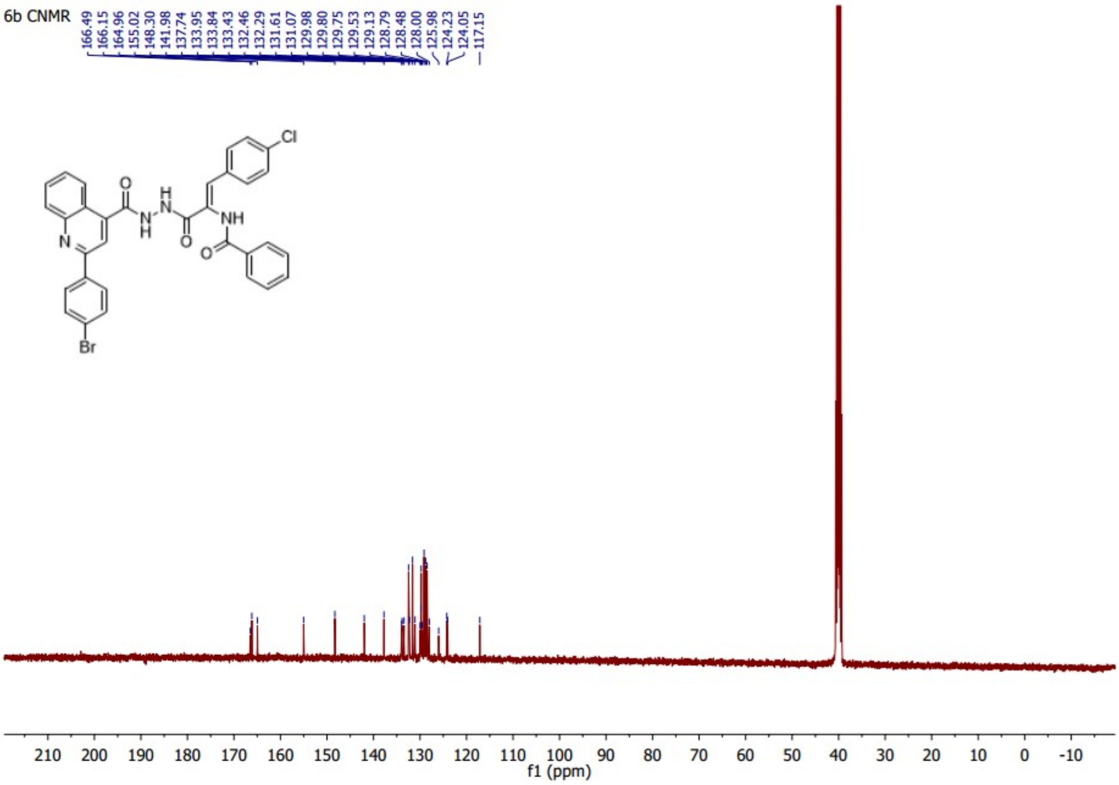

Figure S6: ^{13}C-NMR spectrum of compound **6b**

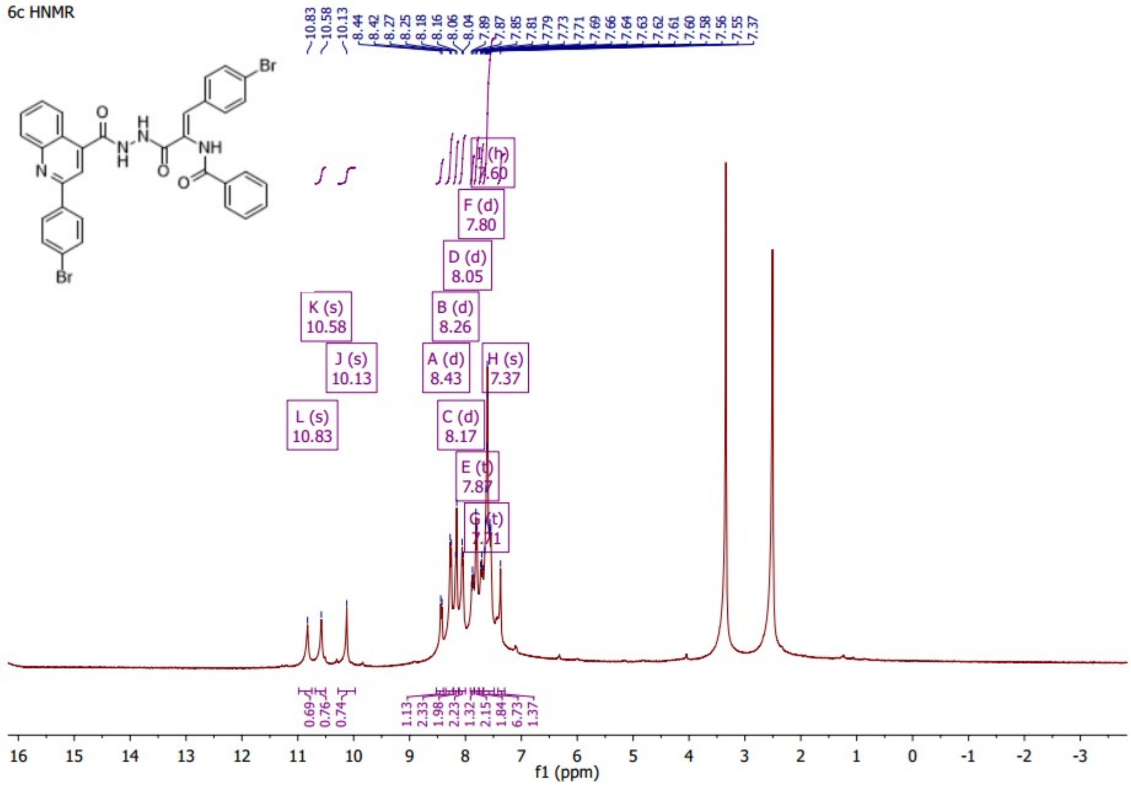

Figure S7: ^{1}H-NMR spectrum of compound **6c**

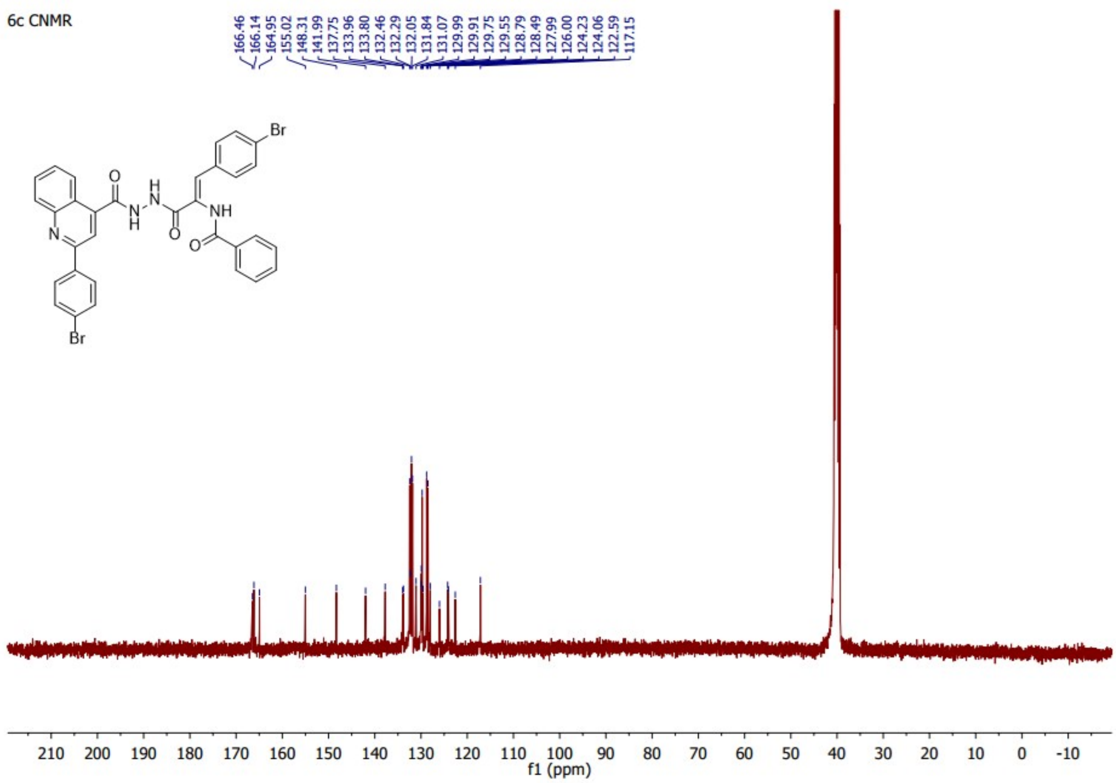

Figure S8: ^{13}C-NMR spectrum of compound **6c**

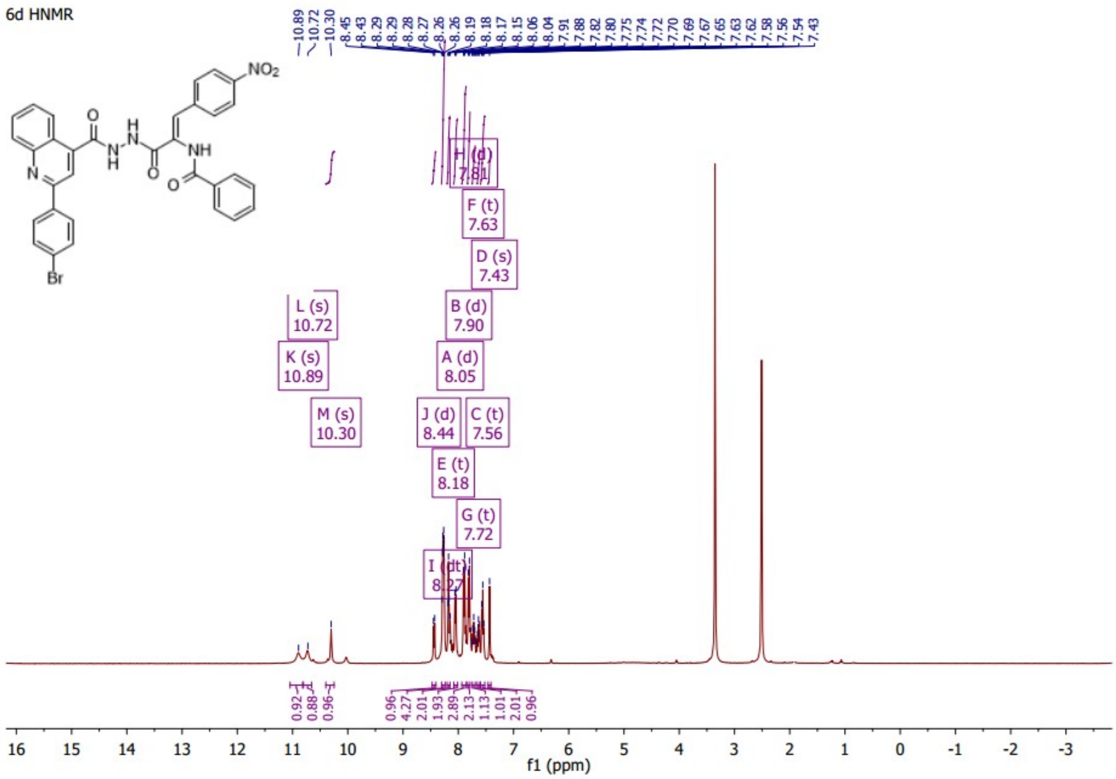

Figure S9: ^{1}H-NMR spectrum of compound **6d**

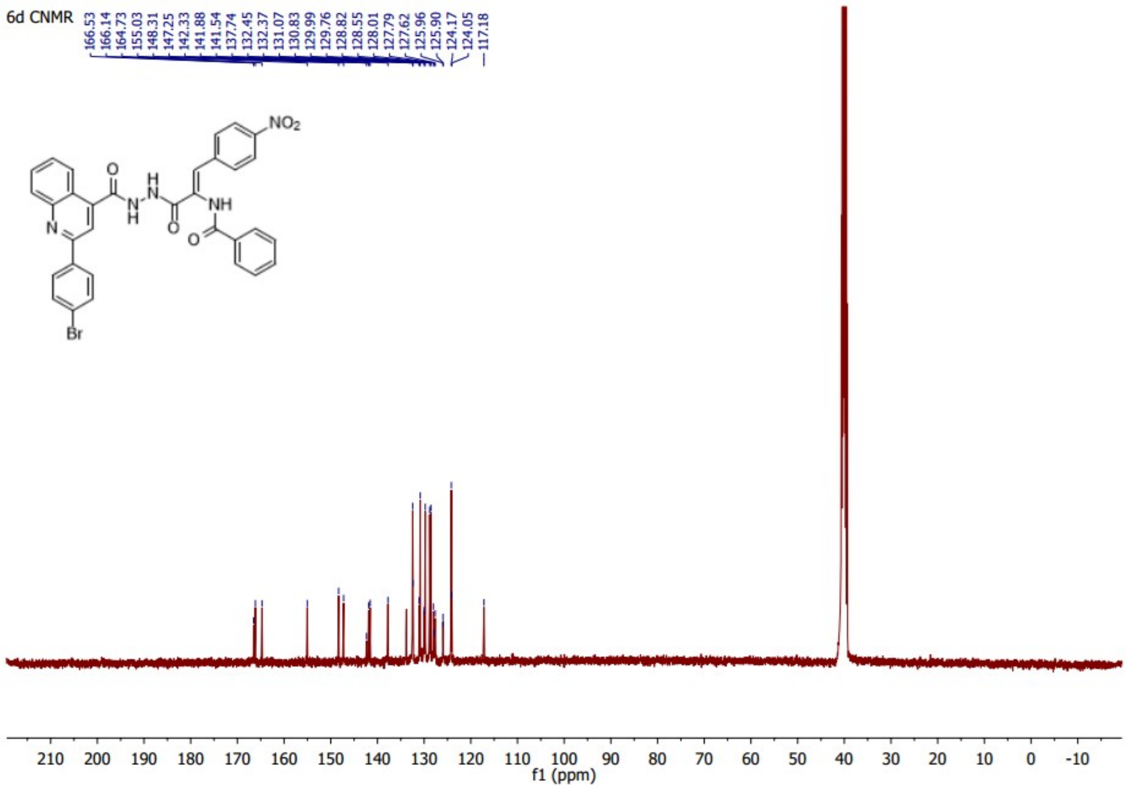

Figure S10: ^{13}C-NMR spectrum of compound **6d**

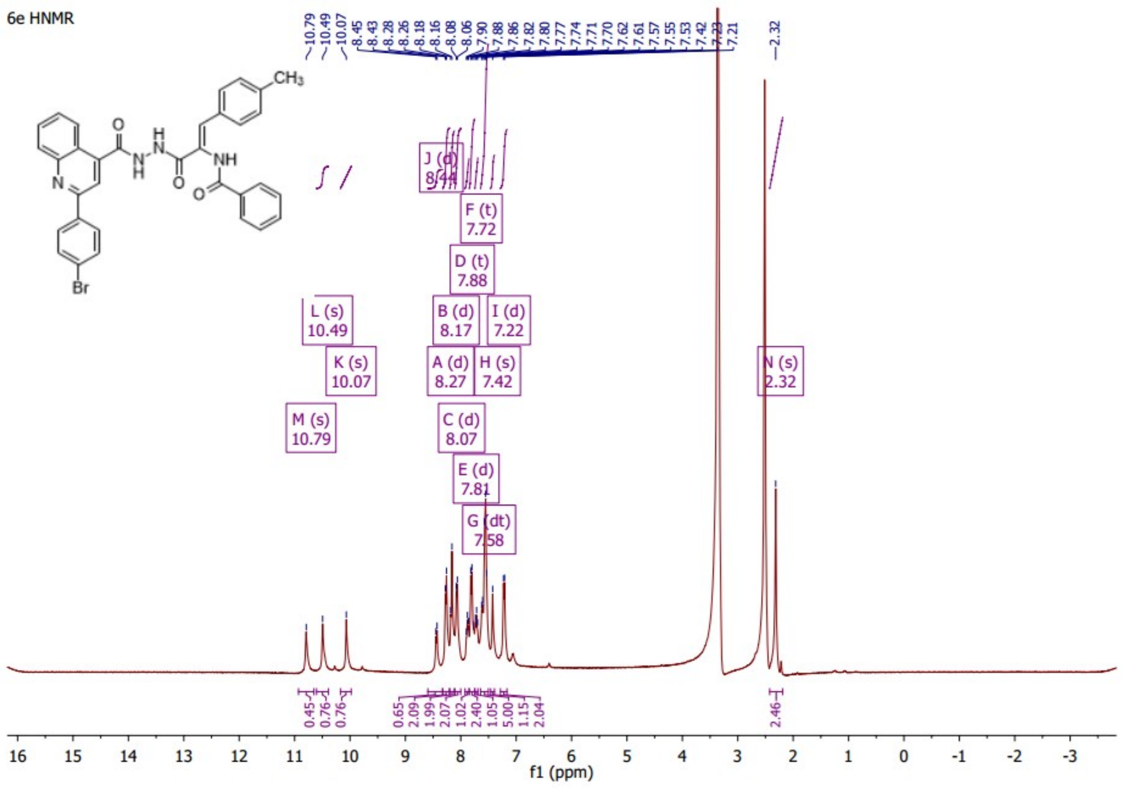

Figure S11: ^{1}H-NMR spectrum of compound **6e**

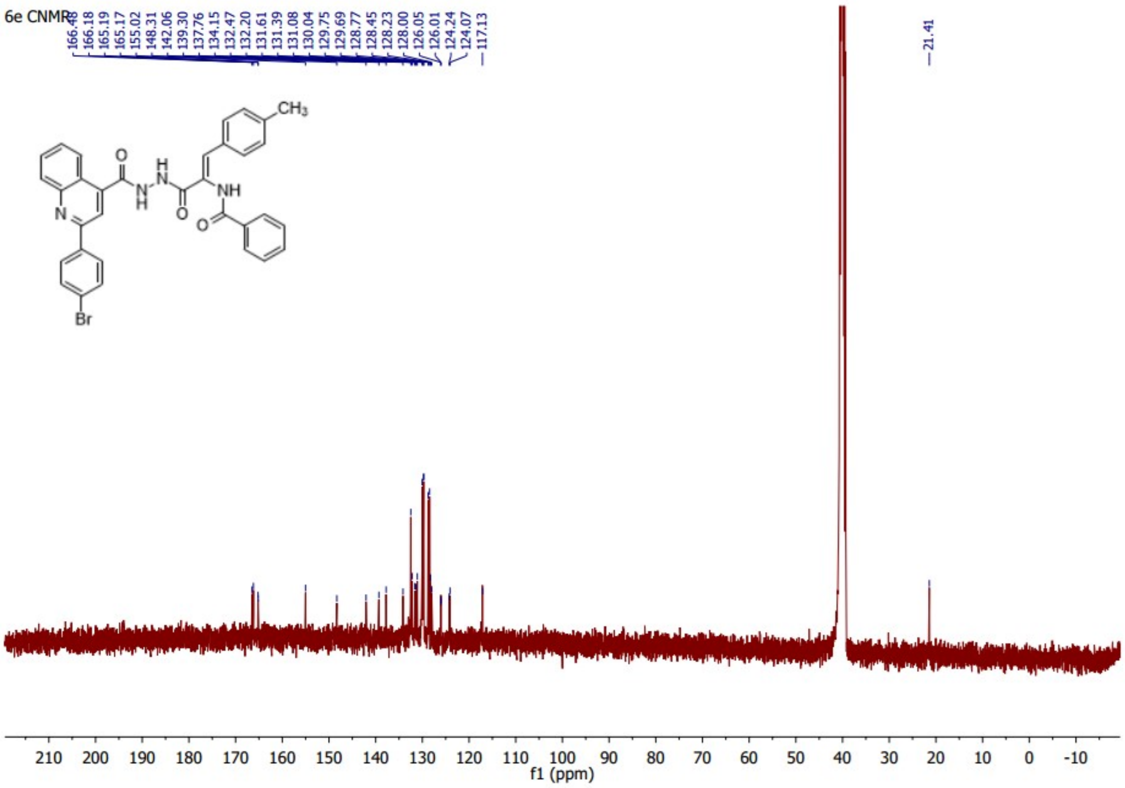

Figure S12: ^{13}C-NMR spectrum of compound **6e**

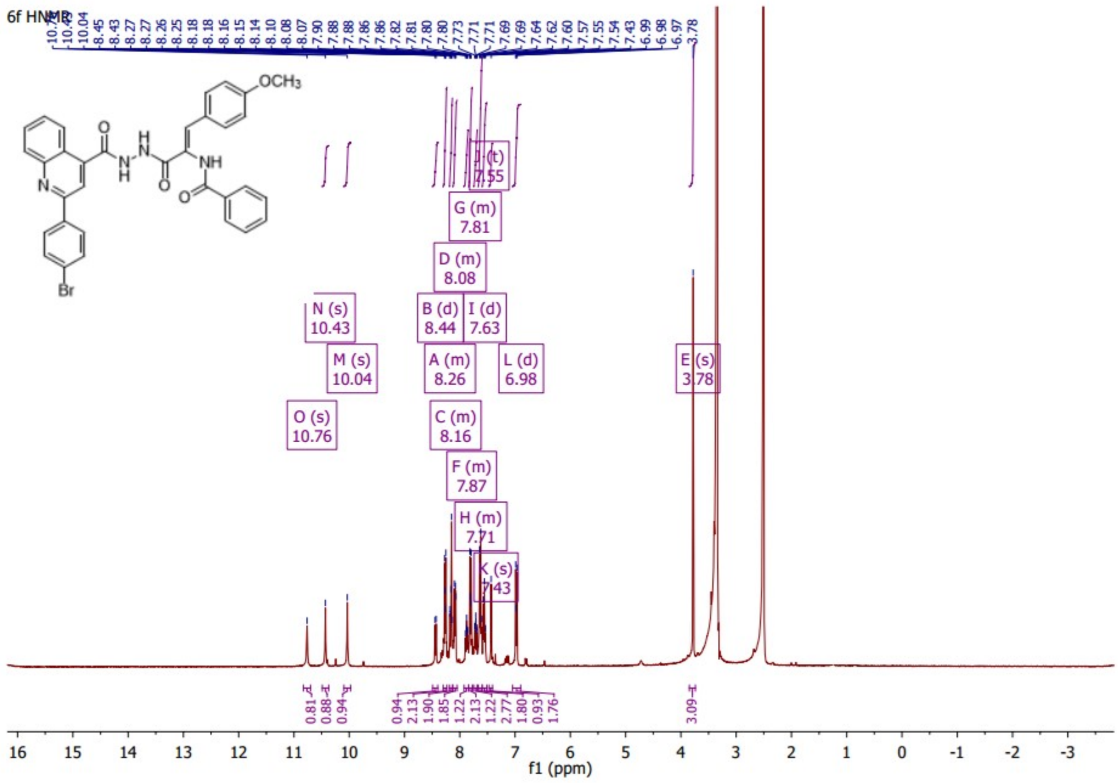

Figure S13: ^{1}H-NMR spectrum of compound **6f**

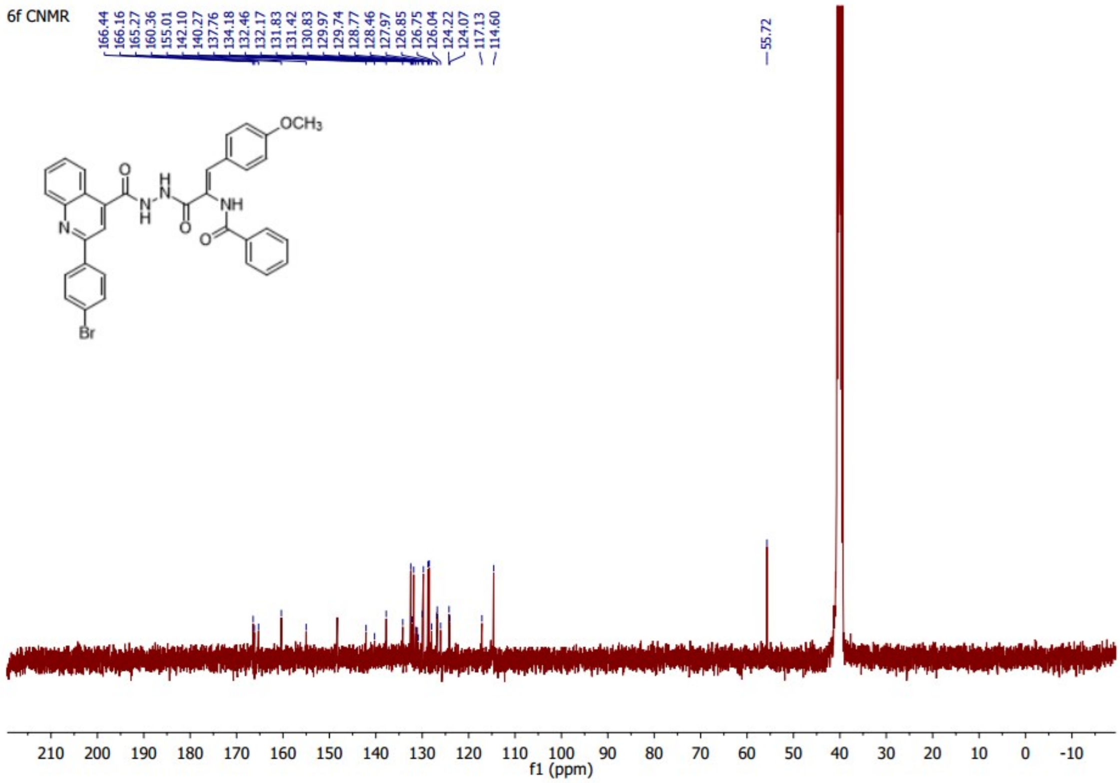

Figure S14: ^{13}C-NMR spectrum of compound **6f**

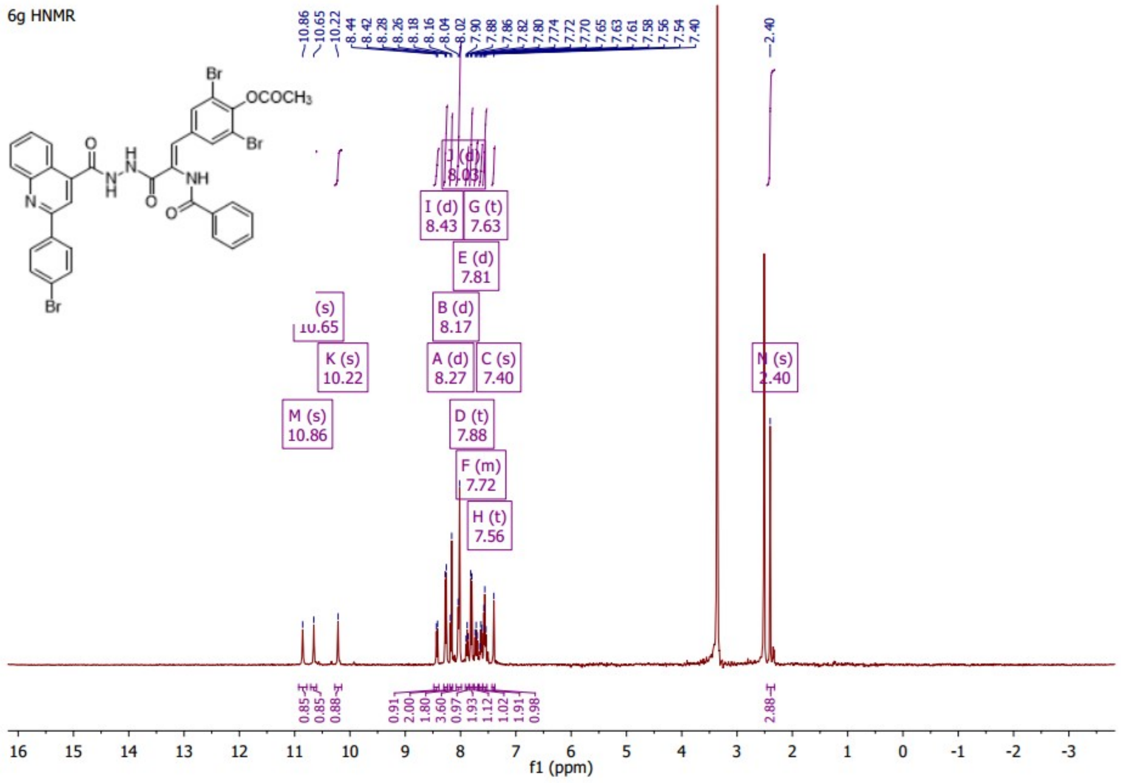

Figure S15: ^{1}H-NMR spectrum of compound **6g**

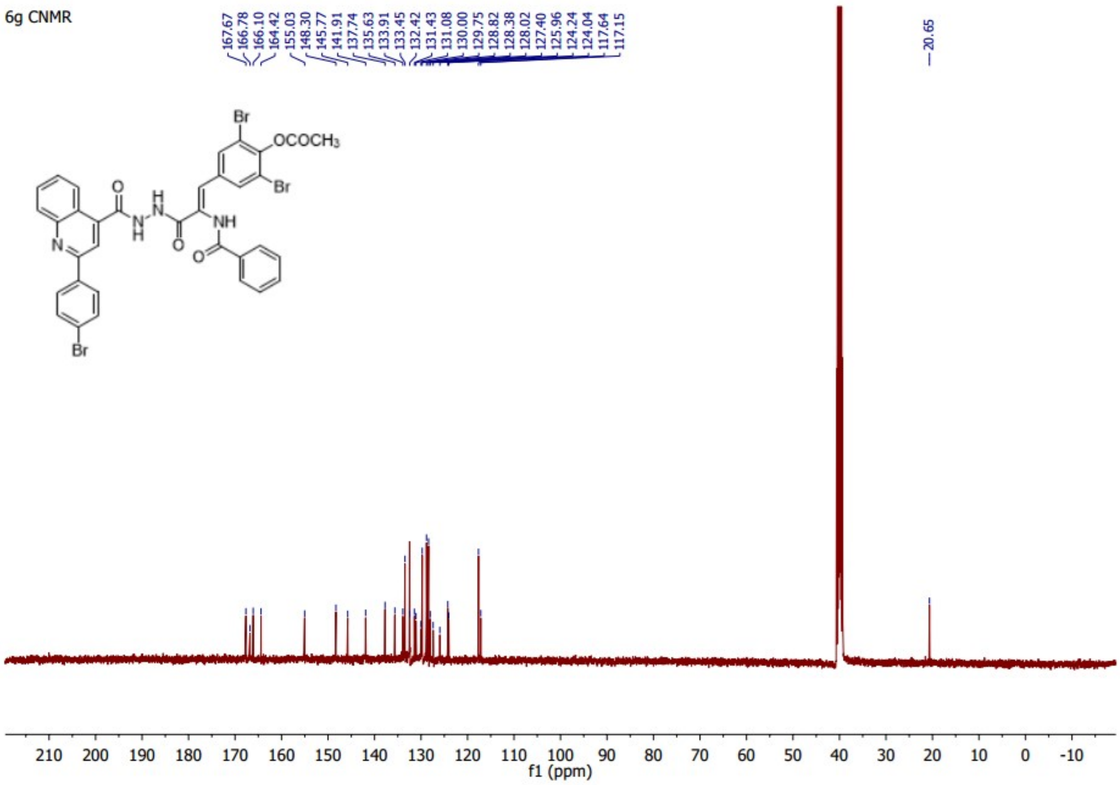

Figure S16: ^{13}C-NMR spectrum of compound **6g**

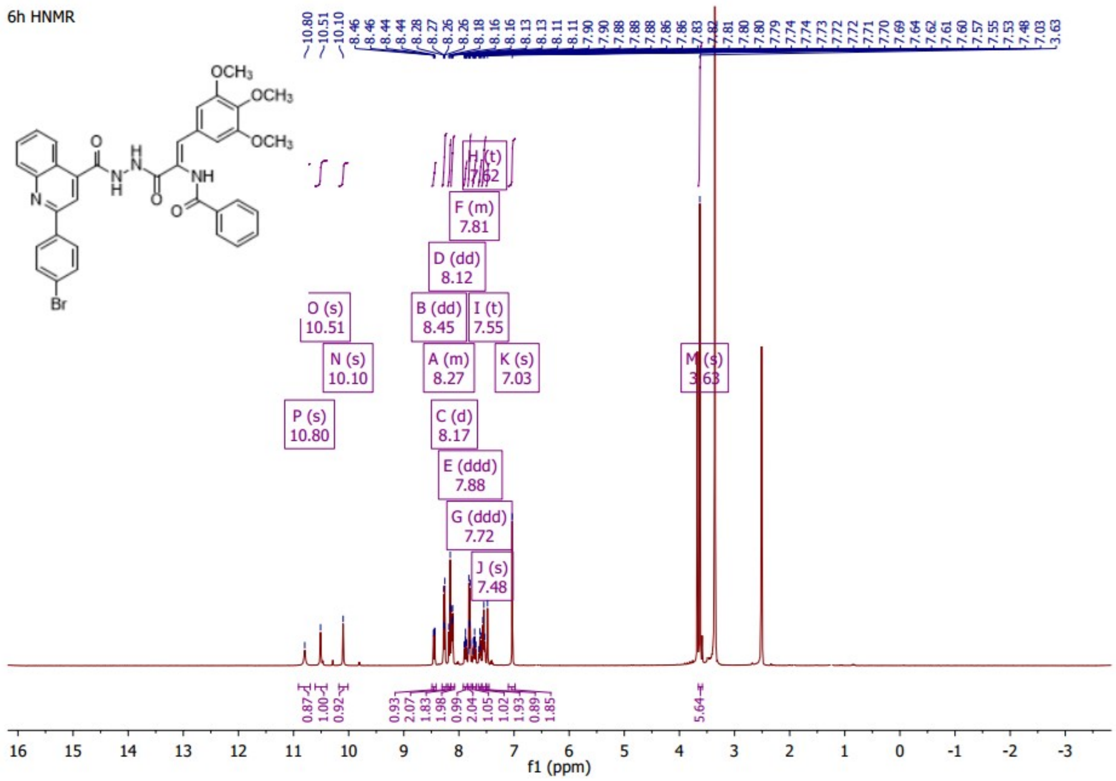

Figure S17: ^{1}H-NMR spectrum of compound **6h**

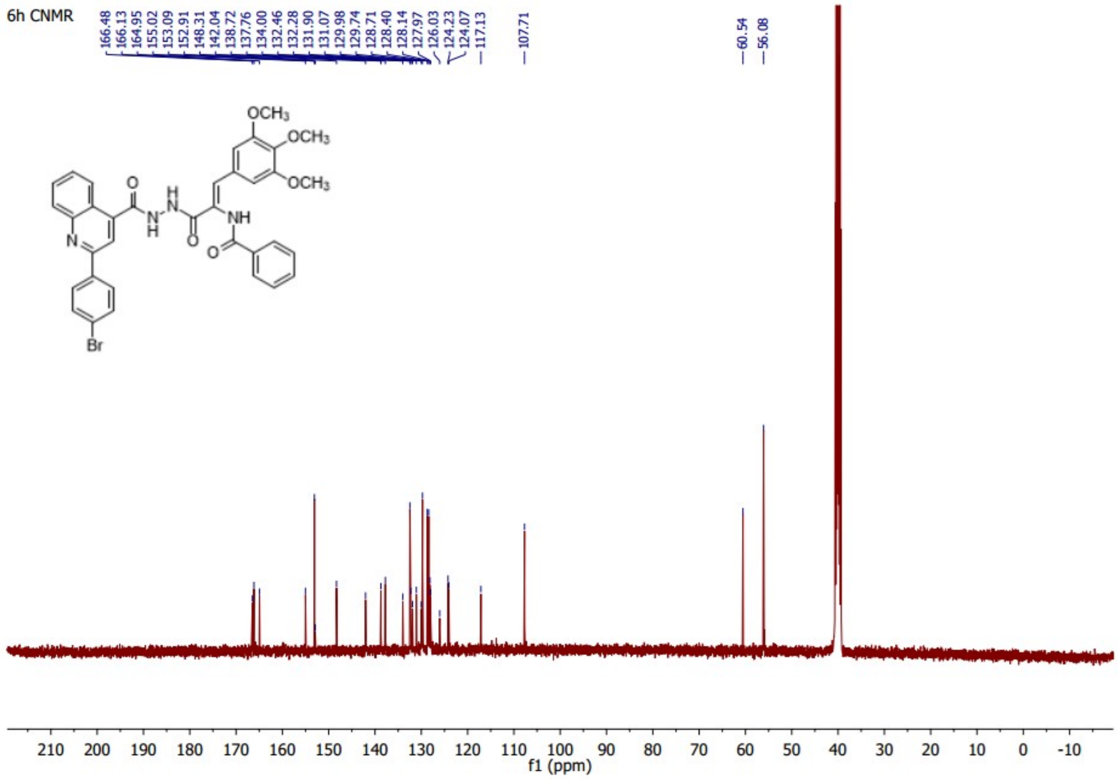

Figure S18: ^{13}C-NMR spectrum of compound **6h**

Appendix A

S4.2. Biological Studies

S4.2.1. Cytotoxic activity evaluation

To measure the cytotoxic actvity of the prepared derivatives **5** and **6a-h** in breast adenocarcinoma (MCF-7) cell line. Cell viability assay was assessed using MTT assay method. Cells at density of 1 x 10^4 were seeded in a 96-well plate at 37 °C for 24 h under 5% CO_2. After incubation, the cells were treated with different concentrations of the test hybrid **5** and **6a-h** and incubated for 24 h, then 20 µl of MTT solution at 5 mg/mL was applied and incubated for 4 h at 37 °C. Dimethyl sulphoxide (DMSO) in volume of 100 µl was added to each well to dissolve the purple formazan that had formed. The color intensity of the formazan product, which represents the growth condition of the cells, is quantified by using an ELISA plate reader (EXL 800, USA) at 570 nm absorbance. The experimental conditions were carried out with at least three replicates, and the experiments were repeated at least three times.

S4.2.2. Cell cycle analysis of compound 6f

Cell cycle analysis in MCF-7 cells was investigated using fluorescent Annexin V-FITC/ PI detection kit (*BioVision* EZCell™ Cell Cycle Analysis Kit Catalog #K920) by flow cytometry assay. MCF-7 cells at a density of 2 × 10^5 per well were harvested and washed twice in PBS. After that, the cells were incubated at 37 °C and 5% CO_2. The medium was incubated with the tested compound **6f** at the IC_{50} (µM) for 48 h, washed twice in PBS, fixed with 70% ethanol, rinsed again with PBS. Afterward, medium was stained with DNA fluorochrome PI for 15 min at 37 °C. The samples were immediately analyzed using *Facs Calibur* flow cytometer (Becton and Dickinson, Heidelberg, Germany).

S4.2.3. Apoptosis assay for compound 6f

Apoptosis in MCF-7 cells was investigated using fluorescent Annexin V-FITC/ PI detection kit (*BioVision* Annexin V-FITC Apoptosis Detection Kit, Catalog #: K101) by flow cytometry assay. MCF-7 cells at a density of 2 × 10^5 per well were treated with compound **6f** at the IC_{50} (µM) for 48 h, then the cells were harvested and stained with Annexin V-FITC/ PI dye for 15 min in the dark at 37 °C. The samples were

immediately analyzed using *FACS Calibur* flow cytometer (Becton and Dickinson, Heidelberg, Germany).

S4.2.4. EGFR kinase Assay

Compound **6f** and Lapatinib were evaluated for their EGFR kinase inhibitory activity according to manufacturer's instructions using # BPS Bioscience *EGFR Kinase Assay Kit* Catalog # 40321.

BPS Bioscience

6042 Cornerstone Court W, Ste B
San Diego, CA 92121
Tel: 1.858.829.3082
Fax: 1.858.481.8694
Email: info@bpsbioscience.com

Data Sheet
EGFR Kinase Assay Kit
Catalog # 40321

DESCRIPTION: The epidermal growth factor receptor (EGFR; ErbB-1; HER1) is the cell-surface receptor for members of the epidermal growth factor family. Overexpression and/or hyperactivation of EGFR kinase is associated with several human cancers such as lung, glioblastoma, and epithelian tumors of the neck and head, leading to the development of anticancer therapeutics targeting EGFR. The *EGFR Kinase Assay Kit* is designed to measure EGFR Kinase activity for screening and profiling applications using Kinase-Glo® MAX as a detection reagent. The EGFR Kinase Assay Kit comes in a convenient 96-well format, with enough purified recombinant EGFR enzyme, EGFR substrate, ATP and kinase assay buffer for 100 enzyme reactions.

COMPONENTS:

Catalog #	Reagent	Amount	Storage	
40187	EGFR (wild type)	2 µg	-80°C	***Avoid multiple freeze/ thaw cycles!***
	5x Kinase assay buffer	1.5 ml	-20°C	
	ATP (500 µM)	100 µl	-20°C	
40217	50x PTK substrate Poly(Glu:Tyr 4:1)	100 µl	-20°C	
	96-well plate, white	1	Room Temp.	

MATERIALS OR INSTRUMENTS REQUIRED BUT NOT SUPPLIED:
Kinase-Glo MAX (Promega #V6071)
Dithiothreitol (DTT, 1 M; optional)
Microplate reader capable of reading luminescence
Adjustable micropipettor and sterile tips
30°C incubator

APPLICATIONS: Useful for studying enzyme kinetics and screening small molecular inhibitors for drug discovery and HTS applications.

STABILITY: Up to 6 months when stored as recommended.

REFERENCE:

Nakamura, J.L. *Expert Opin. Ther. Targets* **11(4)**:463-472 (2007)

Annexin V-FITC Apoptosis Detection Kit

(Catalog #: K101-25, -100, -400; Store at 4°C; Stable for one year)

I. Introduction:

Annexin V Apoptosis Detection Kit is based on the observation that soon after initiating apoptosis, cells translocate the membrane phosphatidylserine (PS) from the inner face of the plasma membrane to the cell surface. Once on the cell surface, PS can be easily detected by staining with a fluorescent conjugate of Annexin V, a protein that has a high affinity for PS. The one-step staining procedure takes only 10 minutes. Detection can be analyzed by flow cytometry or by fluorescence microscopy. The kit can differentiate between apoptosis and necrosis when performing both Annexin V-FITC and PI staining.

II. Kit Contents:

Components	K101-25	K101-100	K101-400	Part Number
	25 assays	100 assays	400 assays	
Annexin V-FITC	125 µl	500 µl	2 ml	K101-XX(X)-1
1X Binding Buffer	12.5 ml	50 ml	2 x 100 ml	K101-XX(X)-2
Propidium Iodide (PI)	125 µl	500 µl	2 ml	K101-XX(X)-3

III. Annexin V-FITC Assay Protocol:

A. Incubation of cells with Annexin V-FITC

1. Induce apoptosis by desired method.
2. Collect 1-5 x 10^5 cells by centrifugation.
3. Resuspend cells in 500 µl of 1X Binding Buffer.
4. Add 5 µl of Annexin V-FITC and 5 µl of propidium iodide (PI 50µg/ml, optional.)
5. Incubate at room temperature for 5 min in the dark.

Proceed to B or C below depending on method of analysis.

B. Quantification by Flow Cytometry

Analyze Annexin V-FITC binding by flow cytometry (Ex = 488 nm; Em = 530 nm) using FITC signal detector (usually FL1) and PI staining by the phycoerythrin emission signal detector (usually FL2).

For adherent cells, gently trypsinize and wash cells once with serum-containing media before incubation with Annexin V-FITC (A.3-5).

C. Detection by Fluorescence Microscopy

1. Place the cell suspension from Step A.5 on a glass slide. Cover the cells with a glass coverslip.

For analyzing adherent cells, grow cells directly on a coverslip. Following incubation (A.5), invert coverslip on a glass slide and visualize cells. The cells can also be washed and fixed in 2% formaldehyde before visualization. (Cells must be incubated with Annexin V-FITC before fixation since any cell membrane disruption can cause nonspecific binding of Annexin V to PS on the inner surface of the cell membrane.)

2. Observe the cells under a fluorescence microscope using a dual filter set for FITC & rhodamine.

Cells that have bound Annexin V-FITC will show green staining in the plasma membrane. Cells that have lost membrane integrity will show red staining (PI) throughout the nucleus and a halo of green staining (FITC) on the cell surface (plasma membrane).

RELATED PRODUCTS:

Apoptosis Detection Kits & Reagents
- Annexin V Kits & Bulk Reagents
- Caspase Assay Kits & Reagents
- Mitochondrial Apoptosis Kits & Reagents
- Nuclear Apoptosis Kits & Reagents
- Apoptosis Inducers and Set
- Apoptosis siRNA Vectors

Cell Fractionation System
- Mitochondria/Cytosol Fractionation Kit
- Nuclear/Cytosol Fractionation Kit
- Membrane Protein Extraction Kit
- Cytosol/Particulate Rapid Separation Kit
- Mammalian Cell Extraction Kit
- FractionPREP Fractionation System

Cell Proliferation & Senescence
- Quick Cell Proliferation Assay Kit
- Senescence Detection Kit
- High Throughput Apoptosis/Cell Viability Assay Kits
- LDH-Cytotoxicity Assay Kit
- Bioluminescence Cytotoxicity Assay Kit
- Live/Dead Cell Staining Kit

Cell Damage & Repair
- HDAC & HAT Fluorometric & Colorimetric Assays & Drug Discovery Kits
- DNA Damage Quantification Kit
- Glutathione & Nitric Oxide Fluorometric & Colorimetric Assay Kits

Signal Transduction
- cAMP & cGMP Assay Kits
- Akt & JNK Activity Assay Kits
- Beta-Secretase Activity Assay Kit

Adipocyte & Lipid Transfer
- Recombinant Adiponectin, Survivin, & Leptin
- CETP & PLTP Activity Assay & Drug Discovery Kits
- Total Cholesterol Quantification Kit

Molecular Biology & Reporter Assays
- siRNA Vectors
- Cloning Insert Quick Screening Kit
- Mitochondrial & Genomic DNA Isolation Kits
- 5 Minutes DNA Ligation Kit
- 20 Minutes Gel Staining/Destaining Kit
- β-Galactosidase Staining Kit & Luciferase Reporter Assay Kit

Growth Factors and Cytokines

BioVisionResearch Products
980 Linda Vista Avenue, Mountain View, CA 94043 USA
Tel: 650-428-0236 • Fax: 650-428-0336 • www.biovision.com
Email: tech@biovision.com

abcam®
discover more

ab139418 – Propidium Iodide Flow Cytometry Kit for Cell Cycle Analysis

Instructions for Use

To determine cell cycle status in tissue culture cell lines by measuring DNA content using a flow cytometer.

This product is for research use only and is not intended for diagnostic use.
